# Graph-Based Generation and Reduction of Complex Chemical Reaction Networks

**DOI:** 10.1021/acs.jcim.6c01309

**Published:** 2026-07-08

**Authors:** Shachar Fite, Zeev Gross

**Affiliations:** Schulich Faculty of Chemistry, 26747 Technion—Israel Institute of Technology, Haifa 32000, Israel

## Abstract

The steep growth of chemical reaction networks, quickly reaching sizes that hinder kinetic analysis, calls for effective reduction methods. Existing methods rely heavily on detailed rate data and are routinely validated only on selected published mechanisms, thus, limiting their scope. We now introduce first a bipartite network generation model developed to reproduce structural and kinetic features of both published and enumerated combustion mechanisms, enabling the large-scale simulation and benchmarking of reduction strategies. Building on this foundation, a topology-based algorithm (MolRank) was developed. It ranks the redundancy of reactions and species, allowing network pruning using only thermodynamic data. Validation across thousands of simulated networks and its application to combustion reaction networks show that MolRank can prune redundant species and reactions on a very large scale. We suggest this framework as a scalable route for testing and applying reduction methods, opening new opportunities for the analysis of combustion, atmospheric, and biochemical reaction systems.

## Graphical abstract

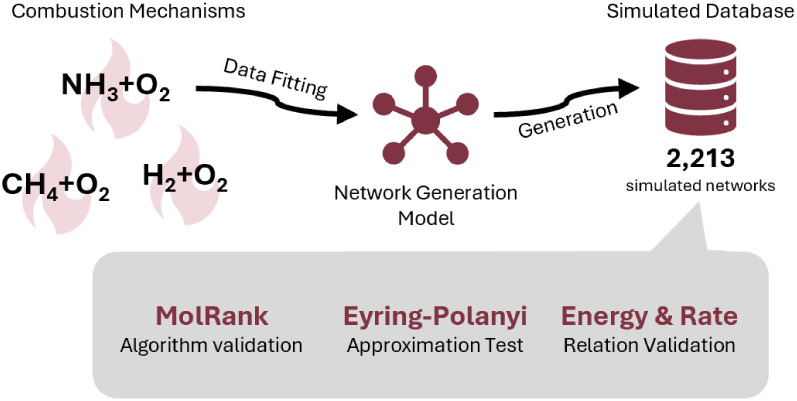

## Introduction

Chemical reactions lie at the core of chemistry, governing how matter transforms under diverse physical and chemical conditions. Chemical reaction networks (CRNs) provide a unifying framework for describing the pathways linking reactants, intermediates, and products in a reaction system.^1,2^ They are naturally represented as bipartite graphs connecting species and reaction nodes and underpin research across combustion, atmospheric, and biochemical chemistry.^3^

Traditionally, CRNs are assembled by expert assignment of elementary steps, validated through experiments and calculations.^4^ The advent of computational chemistry has enabled automated CRN generation from given reactants and conditions, most commonly via elementary reaction enumeration (ERE).^5^ Rule-based ERE methods rely on enumerating reactions from a predetermined set of templates (“rules”).^4,6,7,8^ While efficient, these methods embed strong chemical biases and risk omitting unanticipated pathways. In contrast, graph-based methods attempt systematic, unbiased exploration by enumerating all transformations on molecular graphs.^9,10^ Although more general, these methods suffer from combinatorial explosion, producing highly redundant networks and requiring extensive pruning.^11–14^

CRNs often contain redundant reactions or species that obscure the key mechanistic pathways and hinder downstream applications. Reduction strategies are commonly applied to resolve this issue, but one must consider that they change dramatically depending on the size and information on the network.^15^ The most accurate strategies are quite computationally expensive and require full kinetic information and thus are applicable to relatively small graphs only.^16,17^ The efficacy of a given reduction algorithm is often tested for a specific chemical system, which limits its generality and robustness.

These combinatorial, computational, and methodological challenges call for a complementary perspective: the generation of artificial CRNs to allow rigorous testing and analysis of network structure and reduction algorithms. The construction of random reaction networks, often referred to as *artificial chemistries*,^18^ has been widely used to investigate generic properties of reactive systems, including deficiency,^19^ autocatalysis,^20,21^ dynamics,^22^ and origin-of-life scenarios.^20,23^ Over the years, numerous network generation approaches have been developed,^22,24–27,28^ most of which represent reaction systems as abstract bipartite graphs or hypergraphs and either randomly sample species connectivity or sample from some predefined reaction set. A limited number of software tools automate this process and provide accompanying analysis capabilities for chemical networks.^29,30^ Still, most existing approaches emphasize topological properties, while treating reaction- and species-level attributes—such as thermodynamics and rate constants—only implicitly or not at all, thereby limiting their applicability to kinetic modeling and reduction studies.

We now address these challenges through two complementary developments. First, we introduce a bipartite network representation model for CRNs and apply it to create a large-scale database of 2,213 simulated reaction networks. This enables systematic testing and validating of common network-reduction strategies and kinetic approximations. Second, we present MolRank, a purely graph-based ranking algorithm—analogous to PageRank^31^—to identify and remove redundant species and reactions. MolRank was validated by the simulated database, as well as with more traditional kinetic techniques. An overview of the workflow is shown schematically in Figure 1.

**Figure 1:**
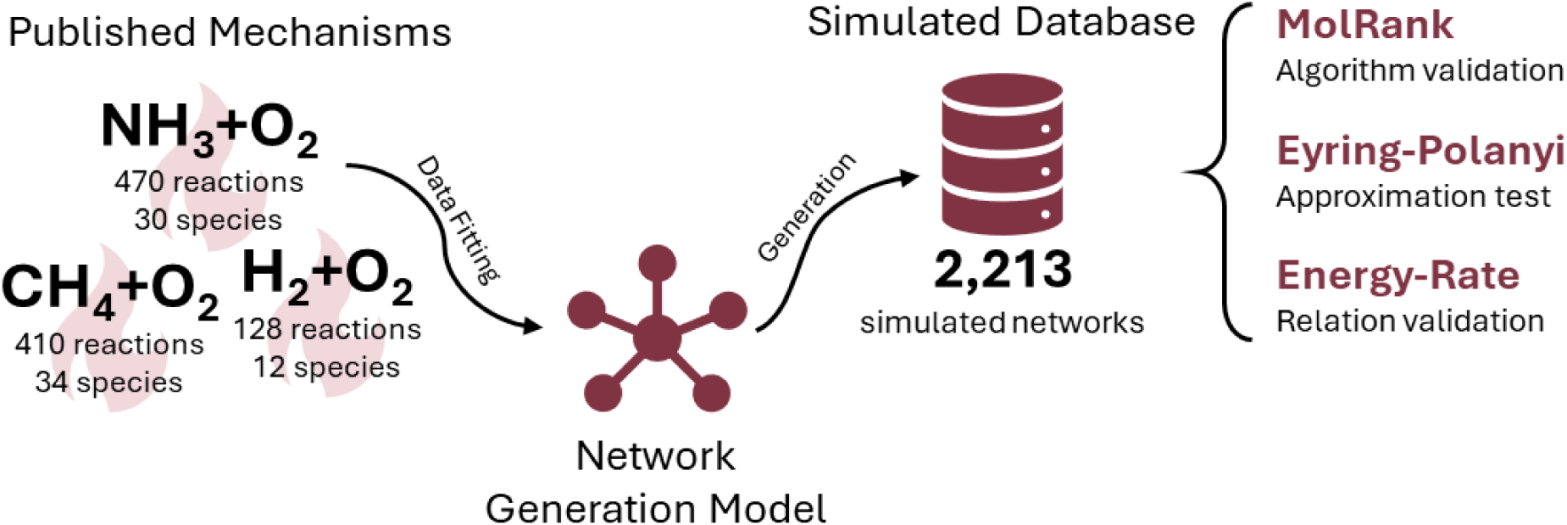
Summary of the study design. Published mechanisms are first used to fit the network-generation model. The fitted model produces a large database of simulated networks with assigned thermodynamics and kinetics. These networks enable testing of common reduction approaches and approximations as well as the MolRank algorithm.

## Methods

This section details the benchmark reaction networks used for fitting the network generation model, the derivation of the network generation model, and the derivation of the MolRank algorithm. Details on the ERE methods used to generate the enumerated networks are presented in the SI.

### Benchmark Reaction Networks

Combustion chemistry was chosen as the primary domain for benchmarking because of its well-defined kinetic behavior: All reactions in these mechanisms follow mass-action kinetics, have a small set of source species, and the sole activating agent is temperature. This simplicity allows for more direct parameter fitting for the network generation. Furthermore, combustion networks are well-studied and experimentally validated,^32,33^ making them an ideal baseline for evaluating both network structure and kinetic fidelity.

Ammonia, methane, and hydrogen combustion mechanisms were selected as benchmarks. Mechanistic sources and details are summarized in Table 1. Each mechanism includes full reaction rate parameters, stoichiometries, and thermochemical data. Prior to use, each mechanism underwent preprocessing to isolate only the subset of reactions reachable from its source reactants. For methane combustion, for instance, only the reaction tree originating from methane and oxygen was retained. This procedure ensured that each benchmark represented a network with known source species, which could be directly compared to networks generated by the ERE protocol and the proposed model.

**Table 1:** Details of Published Networks Used as a Benchmark

			**Number of Reactions**		**Number of Species**	
**Mechanism**	**Reference**	**Source Species**	Original	**Reduced**	Original	**Reduced**
**Ammonia**	^34^	NH_3_ + O_2_	2394	**470**	136	**30**
**Methane**	^35^	CH_4_ + O_2_	624	**410**	53	**34**
**Hydrogen**	^36^	H_2_ + O_2_	138	**128**	15	**12**

On top of the published networks, an enumerated network was made for ammonia combustion. Details of their generation are given in Section 3.1 in the SI, the network has 150,292 reactions and 101 species. Because of the size of these networks, they only have calculated energies for species, without kinetic parameters.

### Network Generation Model

The framework for generating random reaction networks is split into topology and property sampling. The purpose of the topology sampling model is to imitate structural patterns in CRNs, while property sampling is meant to simulate the reaction property distributions in CRNs. This approach allows for the decoupling of kinetic and structural features and the fitting of independent prior distributions for them.

Preferential attachment (PA) has been suggested as the generating mechanism for CRNs in previous works in multiple chemistries,^28,37,38^ due to the “scale free” behavior of known CRNs. Therefore, a PA model was chosen for the topology generation, as it is hypothesized as the most fitting. For reference, a similar Erdős–Rényi (ER) random graph model^39^ was also implemented, offering a contrast in degree distribution and connectivity patterns. This comparison ensured that any observed performance benefits of the modified PA model could not be trivially achieved by a uniform random connection process.

The details of PA and ER generation algorithms are as follows, for a graph of *M* species and *N* reactions:

1. Initialize: Generate *M* species nodes plus a “null” species node, which represents the absence of a reactant or product during sampling. Inclusion of this node enables reactions with fewer than two reactants and/or products by allowing one sampled position to correspond to “nothing”; for example, *A* + Ø → *B* + Ø is equivalent to *A* → *B*, while *A* + *B* → *C* + Ø represents a 2 → 1 reaction. Without the null species node, the sampling procedure would generate only 2 → 2 reaction patterns. Tuning the frequency of the “null” species in the initial species set allows for control of the frequency of different reaction patterns in the sampled network. In this work we added a single null species to avoid specific chemistry fitting.
2. Reactants sampling: Sample 2 species nodes from the graph. In the ER case, the sampling probability is equal for all species nodes. In the PA case, the sampling probability of species *m* is $D_{\mathrm{out}}\left(m\right)/\underset{k}{\sum }D_{\mathrm{out}}\left(k\right)$ , wherein with *D*_out_(*m*) is the number of consuming reactions (*out* degree) of the species.
3. Product sampling: Sample 2 species nodes from the graph (not in the reactants set). In the ER case, the sampling probability is equal for all species nodes. In the PR case, the sampling probability of species *m* is $D_{\mathrm{in}}\left(m\right)/\underset{k}{\sum }D_{\mathrm{in}}\left(k\right)$ , with *D*_in_(*m*) is the number of creating (*in* degree) reactions of a species. Note that the sum is on all species other than the reactants.
4. Add reaction: If the reaction with the sampled reactants and products does not exist in the graph, add a reaction node connecting them.
5. Repeat steps 2–4 until *N* reactions are in the graph.

The methodology above is similar to the one used by Fisher to study reaction network thermodynamics.^28^ Considering that it does not explicitly enforce conservation laws at the level of individual reactions,^40^ the generated CRNs should be viewed as abstract reaction systems rather than fully stoichiometrically closed chemical mechanisms. This choice is consistent with much of the literature on random reaction networks and artificial chemistries,^19,25,26,28^ where reactions are typically generated using topological or combinatorial rules without imposing mass balance. Relaxing the conservation constraints enables the systematic generation of large, diverse network ensembles, which would otherwise be limited by stoichiometric feasibility.^22^ The primary goal of this work is to benchmark network reduction strategies and graph-based importance measures rather than to reproduce detailed chemistry, making nonmass-conserving artificial CRNs a flexible and literature-aligned testbed. A more detailed analysis of the prevalence of nonconserving patterns in the simulated graphs is presented in Section 10 in the SI. While limited to only molar nonconservation, it shows that these motifs tend to contain central reactions in the graph and are therefore retained by the ranking procedures considered here.

Following the topological sampling, an energy value is sampled for all species. The energy value is drawn from a prior distribution of the species’ energies in the benchmark networks (see Section 2.1 in SI) to best represent their chemistry. Based on the species’ energies, a reaction energy is calculated by *E*_r_ = *E*_products_ – *E*_reactants_ for all the reactions in the graph. Arrhenius parameters (*A*, *β*, *E*_a_) are sampled for each reaction, based on its energy and prior distributions fitted to benchmark reactions (see Section 2.1 in SI). This provides an artificial network with all the information required for full kinetic and thermodynamic analysis.

For kinetic and MolRank analyses, source species can be randomly selected for the graphs. In this work, all simulated graphs were sampled with 2 non-null source species. Additionally, graphs for kinetic analysis are assured to be fully reachable from the source. For large-scale analysis, a database containing 2,613 simulated networks, with varying sizes, along with their kinetic results was made. On top of connectivity, all the graphs were assured to have sufficient solver accuracy (e.g., no negative values) and sufficient kinetic participation as detailed in Section 2.2 in the SI together with the full simulation details.

### MolRank Algorithm

The MolRank algorithm was developed to estimate upper bounds on the maximum possible values for concentrations of chemical species and rates of reactions in a chemical reaction network (CRN) without the need for full kinetic simulations. Low values obtained for either of these two variables are strong indicators of redundancy, as discussed above. Direct estimation of the maxima would normally require complete kinetic parametrization and numerical integration of large systems of ordinary differential equations, which is infeasible for large networks. MolRank circumvents this limitation by providing upper-bound estimates using a graph-based traversal procedure.

Conceptually, the method is inspired by the PageRank algorithm^31^ and related network diffusion models but with adaptation to the flow of chemical concentration. The basic idea stems from the mass-action definition of reaction rate as *k*[*A*]­[*B*] (for a reaction with *A* and *B* reactants), then trivially *k*[*A*]­[*B*] ≤ *k*[*A*]_max_[*B*]_max_, where [*A*]_max_,[*B*]_max_ are the maximal achievable concentrations of *A* and *B*. The estimation of this maximal concentration is at the heart of MolRank.

To demonstrate the approach of maximal concentration estimation of species, we will use the following simple reactive system of $A\overset{k_{1}}{⃗}B,A\overset{k_{2}}{⃗}C$ . For *B* and *C*, it can be estimated by assuming a full reaction of A after *t_A_* = *A*/(*k*_1_ *A* + *k*_2_ *A*), where *A* is the maximal concentration of *A*. The concentration flux to each reactant will be given by the maximal reaction rate times the reaction time, i.e., *f*_1_ = *k*_1_ *At_A_* and *f*_2_ = *k*_2_ *At_A_*. The maximal concentrations of *B* and *C* are derived from the fluxes. This procedure can be repeated for the consuming reactions of *B* and *C*, until all the network is covered.

In the case of multiple reactants, the reaction time for each flux is taken to be the minimum reaction time for each species. For a reaction of type *A* + *B* → *C*, the flux will be *f* = *kAB*min­(*t_A_*,*t_B_*). This way a general reaction graph can have a MolRank score. This approximation holds well for many network topologies but can fail on some, such as networks containing mixed fast equilibrium reactions with slower irreversible reactions. In such situations, the species flux may be dominated by the reversible exchange, while MolRank can overemphasize the larger forward rate constants of the equilibrium pair. Analysis of these motifs (Section 9 in the SI) showed that they were absent in the simulated networks and occurred only rarely in the published combustion mechanisms examined here (3 species in the hydrogen network, 5 in ammonia, and 3 in methane), indicating that this limitation is relevant in principle but not dominant for the systems considered in this study.

More concretely, the algorithm proceeds as below and is described schematically in Figure 2:

1. Initialization: The CRN is represented as a bipartite directed graph of species and reactions. All species are initialized with zero concentration, except for designated “source species” which are given a unit concentration. All reactions are initialized as unvisited with zero rate and zero flux.
2. Iterative Expansion: At Each Iteration:
  All unvisited reactions whose reactants are present in the current set and which have not yet been visited are identified.
  Compute the reactions’ rates by mass-action kinetics. Mark these reactions as visited.
  For each reactant, estimate the “reaction time”—its concentration divided by the sum of rates of reactions that consume it in this step.
  Compute each reaction’s flux as the product of its rate and the minimum “reaction time” across its reactants.
  For each product species (predecessors of the reaction nodes), we update its concentration as the sum of fluxes of reactions producing it. If this concentration exceeds its previous value, consuming reactions (predecessor nodes) are marked as unvisited (since they may now achieve higher rates in a subsequent pass).
  Product species (predecessors of reaction nodes) are added to the reactant set.
3. Termination: The procedure is repeated until all reachable reactions are visited.

**Figure 2:**
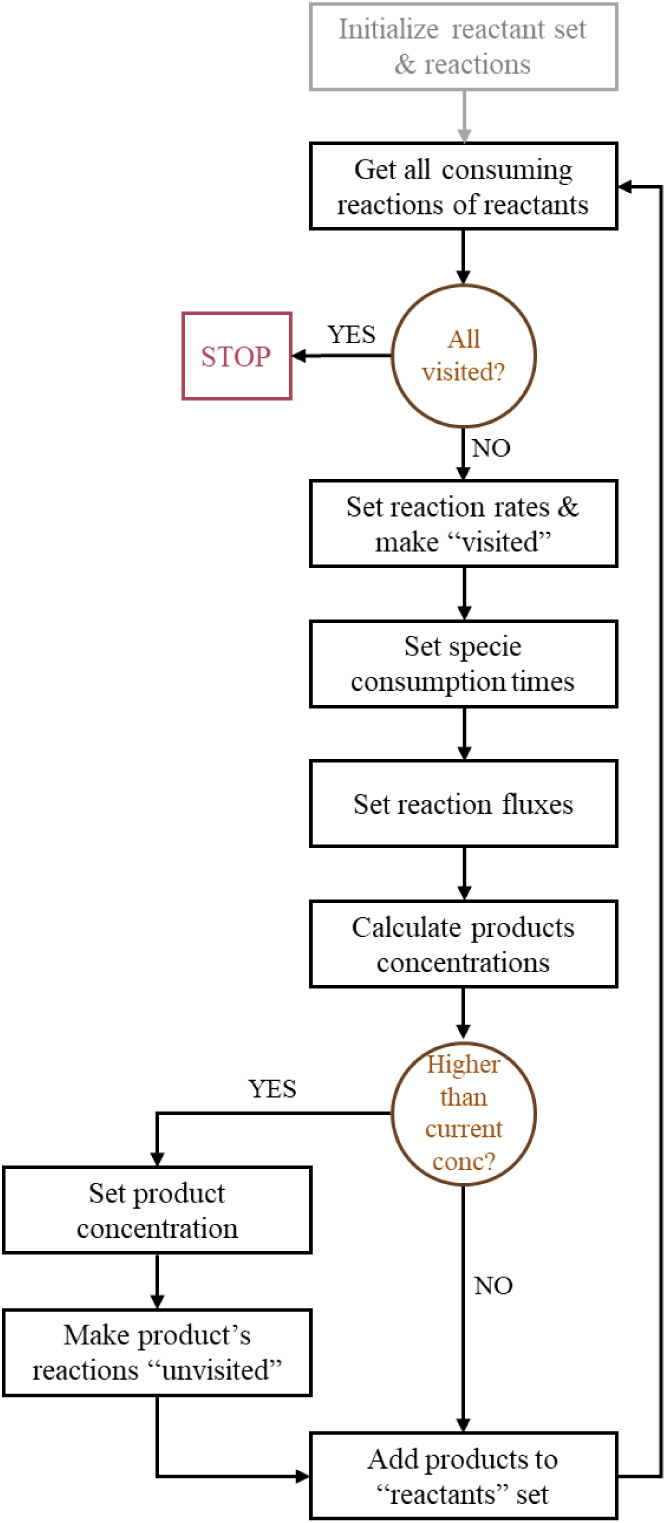
Schematic description of the MolRank algorithm. According to the outline of initiation, iterative expansion, and termination described in the main text.

## Results and Discussion

The performance and applicability of the proposed network generation model were evaluated by comparing its structural and kinetic properties to those of both real and fully enumerated reaction networks. Analysis begins with a quantitative assessment of the degree distribution, reaction energetics, and reaction rate constant distributions, using the Erdős–Rényi (ER) random graph as a structural baseline. We further examine the ability of the model to reproduce the maximal rate distributions. Finally, we assess the MolRank-based reduction approach, demonstrating its capability to identify redundant species and reactions in simulated and published networks.

### Validation of Network Generation Model

#### Published Networks

To evaluate how well the generated networks reproduce real chemical systems, we compared their topological and kinetic characteristics to those reported for published ammonia, methane, and hydrogen combustion mechanisms (see Methods). An Erdős–Rényi (ER) random graph with the same number of species and reactions served as a structural baseline to compare with the preferential attachment model. Model distributions were based on 10 repetitions for each number of species and reactions.

The *degree* of a node (reaction or species) describes its number of connections.^41^ *In-degree* measures the number of incoming edges, e.g., the number of reactions producing a species or the number of reactants in a reaction. *Out-degree* measures the number of outgoing edges, e.g., the number of reactions consuming a species, or the number of products in a reaction. The total degree is the sum of both and reflects the overall chemical connectivity. Agreement between the degree distributions of simulated and reference networks is a paramount metric for validating the topological fitness of the model.

Figure 3 displays the *species degree distributions* as histograms of the number of reactions consuming (out degree), creating (in degree), or both (total degree) per species. The reference networks (in red) exhibit a right-skewed distribution with many low-degree species and a few highly connected hubs. Our model (in blue) reproduces this skew and hub presence, while the ER baseline (in orange) shows a narrow, symmetric peak that fails to capture the heterogeneity of the real reaction connectivity. For the hydrogen network, a noisier reference distribution is obtained due to the smaller number of species in the network, leading to a less apparent fit. These qualitative results are supported by a formal Kolmogorov–Smirnov (KS) goodness-of-fit test^42^ (Table S3), disclosing a statistically significant fit (*p* > 0.05) for the preferential attachment model compared to the ER model, for all cases including hydrogen.

**Figure 3:**
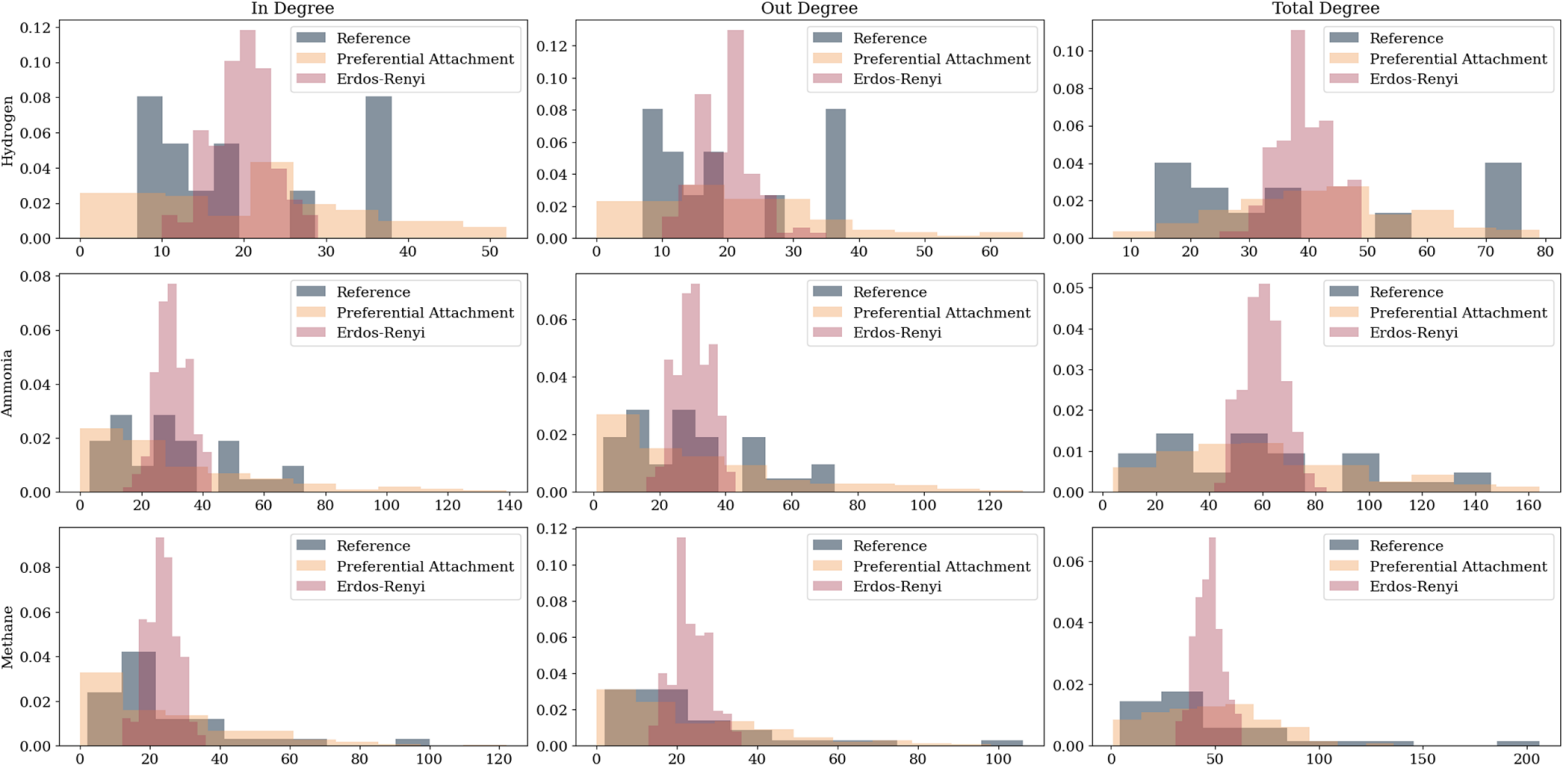
Histogram of species degrees (in, out, and total) for the network, our model-generated network (blue), and an ER random graph baseline (orange) for all three reference networks (red).

*Reaction degree distribution* shows a clear over-representation of reactions with 2 reactants and 2 products in both generating models (“2 → 2” reactions) (Figure 4). This stems from the initial number of “null species” in the graph that can be tuned to fit for any given network. This was not done here to prevent bias for specific chemistry. Additionally, as the sampling assumes a maximum of 2 reactants and 2 products, reactions with a higher number of species involved are not represented. The ER model has a larger (and closer to reference) representation of 1 → 2 and 2 → 1 reactions compared to the preferential attachment model. Thermodynamic realism was assessed via the distribution of reaction free energies (Figure 5), plotted as histograms of Δ*G* values. For both models, the energies match the published distribution in both spread and central tendency, capturing the broad range from strongly exothermic to strongly endothermic steps.

**Figure 4:**
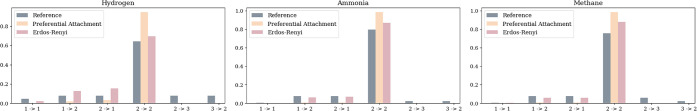
Reaction degree distribution presented by frequency per “reaction type”, defined as (# reactants) → (# products).

**Figure 5:**
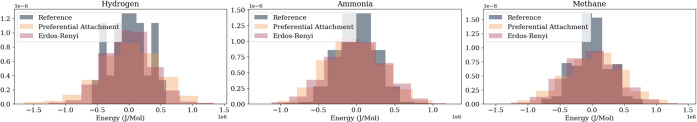
Reaction energy distribution for each of the reference and model-generated networks.

Kinetic property description was addressed by comparing the distribution of logarithmic rate constant values (Figure 6). While the model fit is not perfect, it still reproduces the heavy-tailed nature of the published distribution. It is hypothesized that the relatively large difference stems from our decision to use a simple linear model in the sampling of the activation energy. Although a more sophisticated model could have been used, this was not done to avoid overfitting to a particular chemistry. Accordingly, the maximal reaction rate distribution shows a subpar fit, but again, with heavy-tailed nature and range matching (Figure S8).

**Figure 6:**
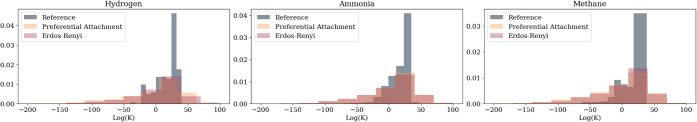
Logarithmic rate constant distribution for reference and model-generated networks.

Overall, the preferential attachment model captures the structural aspects of the published reference mechanism better than random graph baselines, providing evidence that the generation procedure captures the essential features of real chemical reaction networks. The properties of reactions (energy, rate constants) are heavily influenced by the parametrization of prior distributions and are thus relatively similar between random and preferential models. Accordingly, the maximum reaction rate is distributed similarly in both models.

#### Enumerated Networks

For the fully enumerated ammonia combustion network, the degree distribution of species shifts markedly compared to the published mechanism. Instead of the highly tailed profile with prominent hub species, the distribution becomes more symmetric and centered around a characteristic degree value. This change likely arises from the exhaustive inclusion of all stoichiometrically allowed reactions within the enumeration constraints, which suppresses extreme connectivity in favor of more uniform species participation. As a result, the preferential attachment (PA) model—which assumes a “rich-get-richer” growth process—becomes a less accurate descriptor of the connectivity pattern (Figure 7). ER networks at this size show a greatly concentrated degree distribution with a similar typical degree as the reference network. This suggests that the exhaustive enumeration of reactions yields a network structure in-between a preferential attachment to a random graph model (Figure S9).

**Figure 7:**
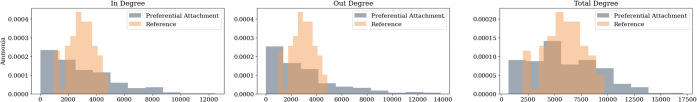
Histogram of species degrees (in, out, and total) for the network, the model-generated network (blue), and the reference enumerated ammonia network (orange). Areas of overlap appear in brown.

In contrast, reaction degree distributions—defined here by the number of reactants and products in each reaction—show much better agreement between the enumerated network and the PA model. The most striking feature, present in both, is the clear dominance of 2 → 2 reactions, which outnumber all other stoichiometries by a wide margin (Figure 8, right). Thermodynamically, comparison to calculated reaction energies reveals that the enumerated and simulated PA networks share the same overall “scaled” energy distribution (Figure 8, left): both exhibit an approximately normal shape centered near zero, though the PA networks have a noticeably larger standard deviation.

**Figure 8:**
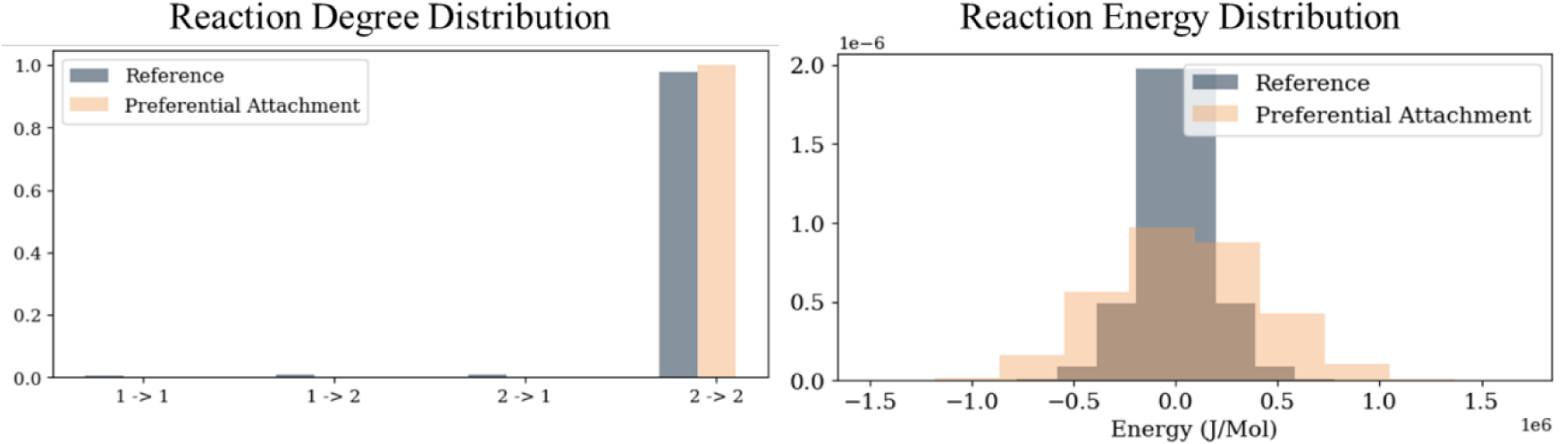
Reaction degree and energy distributions of simulated graphs and enumerated ammonia combustion networks.

Taken together, these results indicate that while the PA-based generation model reproduces the reaction degree and energetics of enumerated networks, it fails to capture the species degree distribution. This discrepancy highlights an inherent limitation of the present generation model when applied to fully enumerated mechanisms. Nevertheless, most reduction strategies developed for enumerated reaction networks rely primarily on reaction thermodynamics and kinetics, rather than on detailed graph topology.^1,11,13^ From this perspective, simulated networks that reproduce energetic and kinetic statistics may still provide a useful test bed for evaluating reduction techniques. This could be followed by the development and validation of generation models that simultaneously reproduce both topological and kinetic features of fully enumerated networks, which are beyond the scope of this work.

### Simulating Common Network Reduction Techniques

Using the network generation model, large-scale enumeration of 2,613 reaction graphs (see Methods) enables us to explore phenomena that are rarely accessible in real data sets. These simulated networks are an ideal testbed for evaluating commonly used network reduction methods, since they are unbiased, internally consistent, and large. Common network reduction techniques for enumerated networks are evaluated in this section. This serves as a demonstration of the new capabilities provided by the network generation model as well as a benchmark for the validation of the MolRank algorithm.

The maximum reaction rate and maximum species concentration are used to quantify reaction and species redundancy, respectively. They are obtained trivially from the kinetic simulation—making them relatively easy to calculate even for larger networks, unlike other common redundancy metrics^15^ that require preselected target species, which are unusable for simulated networks. The underlying assumption is straightforward: if a cheaper-to-compute descriptor reproduces a good upper bound for maximal rates or concentrations, it can serve as a proxy for full kinetic simulations and enable efficient network reduction. Out of the redundancy metrics, thermodynamic ones such as reaction energy and species shortest-energy path are the most frequently used.^10,11,13^ They are very useful for network reduction at scale: cheap to calculate and do not require kinetic integration. Intuitively, reactions with high energy tend to proceed slowly, and species at higher energy relative to precursors tend to accumulate only weakly.

Testing this intuition in the simulated database reveals a clear relationship between the reaction energy and the maximal reaction rate. Figure 9A reports, for each energy value, the 95th percentile of maximal rates among all reactions with equal or higher energy. This presentation exposes the trend immediately: endergonic reactions exhibit progressively smaller maximal rates, and a roughly linear decay is observed over a substantial portion of the positive energy region. This metric has a limited reduction effect, considering that only about half of all simulated reactions have positive energies.

**Figure 9:**
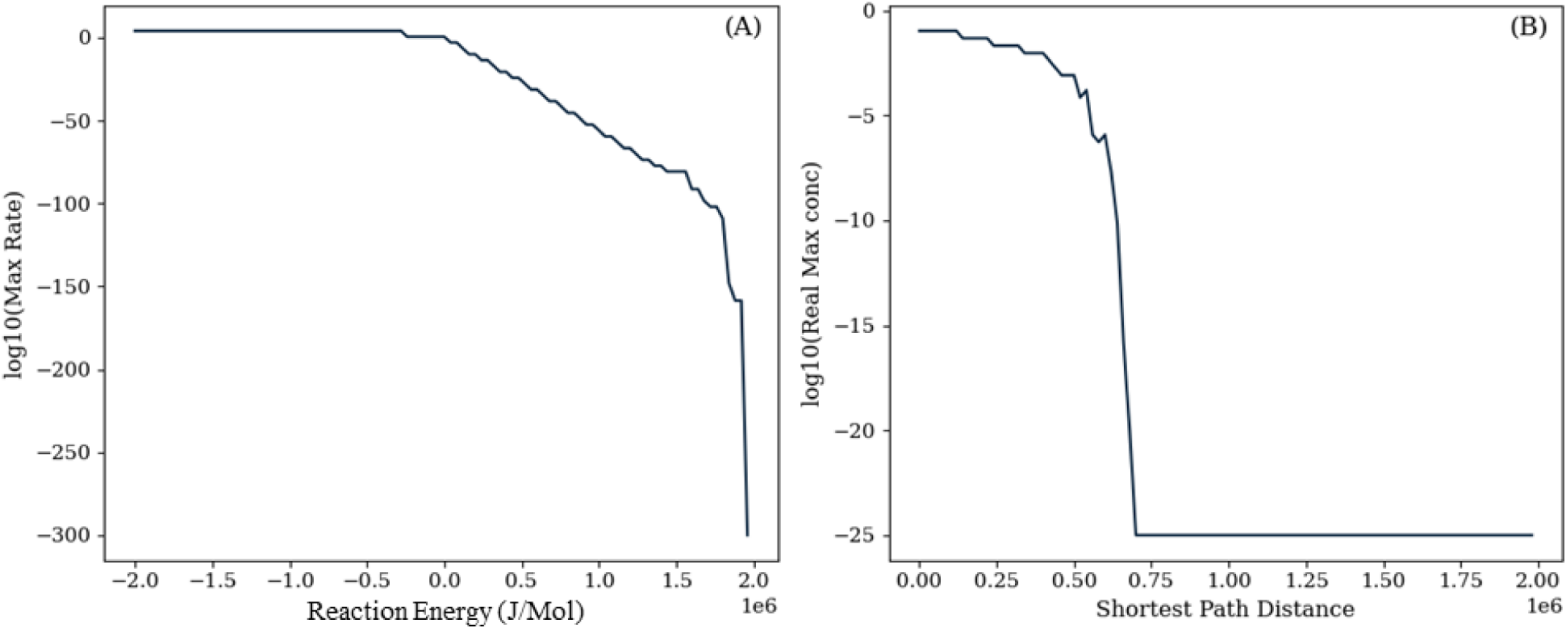
95th percentile of log­(Max rate) (A) or log­(Max Concentration) (B) of all reactions or species in the database with energy equal to or higher than a given threshold.

A corresponding analysis for the shortest-energy-path descriptor (Figure 9B) shows a contrasting, step-like pattern. The 95th-percentile maximal concentration drops sharply once the species’ energy exceeds a certain threshold, indicating that sufficiently high-energy species consistently reach negligible concentrations. Although this trend confirms the value of the shortest-path metric, it applies only to the small fraction (∼1%) of species that occupy the high-energy tail of the distribution in the simulated networks. The full scatter-correlation plots for both metrics, together with analogous results for the published mechanisms, are provided in the SI.

The Eyring–Polanyi (EY) approximation^43,44^ is commonly applied to estimate reaction rates and species concentrations when full kinetic data is missing.^11^ Using the EY approximation, reaction rate constants are estimated from activation energy only or by reaction energy given a scaling relation. The accuracy of rate and concentration estimates was tested using the simulated database. It was found that while the correlation between approximated rates and concentrations is quite low, the approximation consistently provides an upper bound for real rate and concentration (Figure 10). The bound is most accurate for the fastest processes, which are also the most chemically important. Eyring–Polanyi is therefore a reliable redundancy descriptor, although it requires kinetic integration and becomes computationally demanding for very large networks. These trends are generally mirrored in published networks (Figure S11). In these systems, the EY approximation remains effective, although its quality as an upper bound is somewhat diminished compared with the simulated graphs. This is probably due to errors stemming from the scaling relation fit in published graphs compared to the ideal simulated graphs.

**Figure 10:**
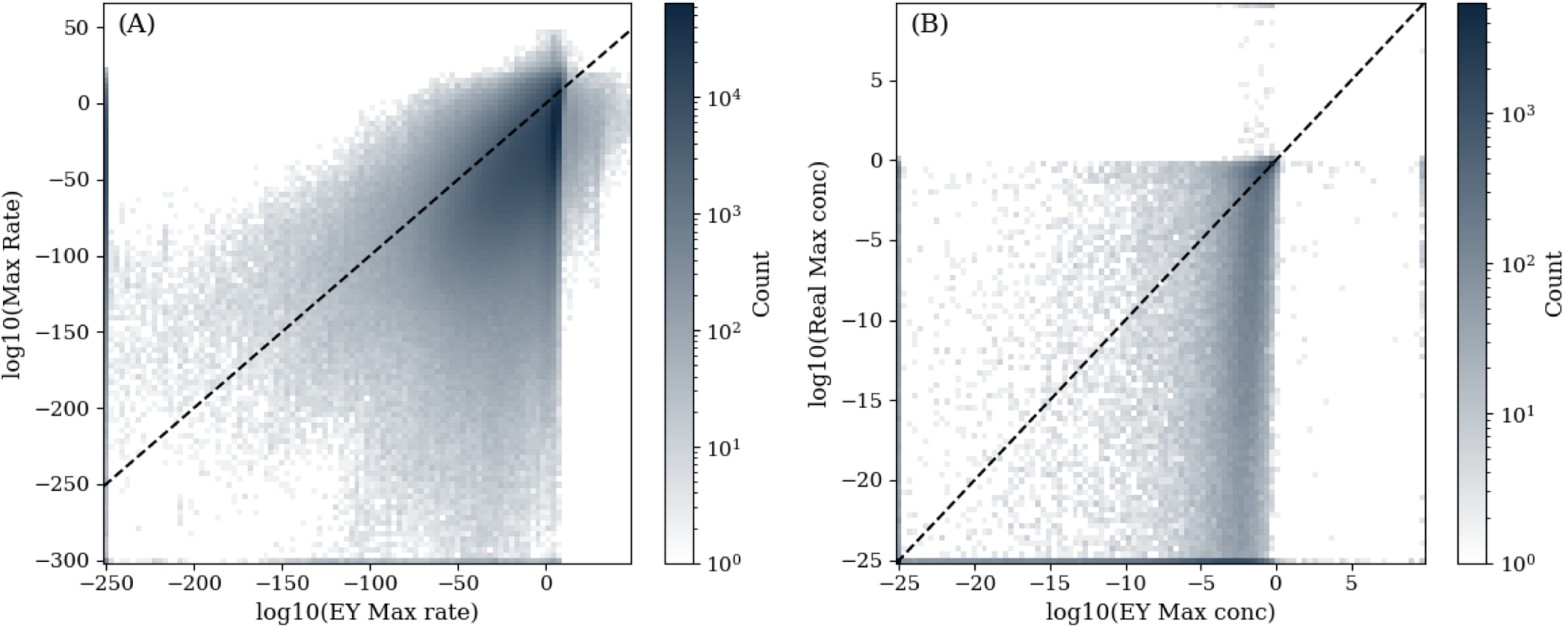
Correlation of (A) maximal reaction rate and (B) maximal concentration estimated by Eyring–Polanyi approximation. Dotted lines are *x* = *y*, guiding the eye for the upper bound quality.

### MolRank Algorithm

The accuracy of MolRank was assessed using three complementary validation strategies designed to capture its accuracy and robustness on multiple network scales. First, simulation-based validation is presented, which allows rigorous comparison between the MolRank-predicted values and the ground truth. Second, starting from published networks, reactions and species were systematically removed by MolRank score; and the resulting kinetic errors were quantified, directly validating the qualities of the reduced network. Third, preserved reactions and species analysis was performed on the ammonia combustion enumerated network to evaluate the method’s ability to retain the relevant chemistry contained in experimentally derived mechanisms after reduction.

#### Simulation-Based Validation

Based on the simulated reaction database detailed above, MolRank rates and concentrations were evaluated in each simulated network using (i) the true rate constants available from the simulation, and (ii) Eyring–Polanyi (EY)-based estimates derived from reaction energies, which represent a more realistic approach in practical applications. These simulated graphs allow for a comparison between the estimated maximum rate and concentration by MolRank to the actual values to directly test the quality of the MolRank analysis.

For the maximum reaction rate (Figure 11A,B), we observe a clear and somewhat surprising trend: EY-based MolRank scores provide a better bound on the true maximum rate than the MolRank computed directly from the known rate constants. This is explained by the systematic overestimation of the EY-based rate constants (Figure 10), leading to higher MolRank rates that are more likely to bind the real rate values. In both cases, for higher rates, points are predominantly located below the *x* = *y* reference line, as exemplified by the red dashed line, representing the 95th percentile of rates of reactions with MolRank scores greater than *x*. In lower rates, the red line exceeds the black line, signifying worse bounding of the MolRank score. Both show that MolRank generally acts as an upper bound, particularly for high-rate reactions, where it is most relevant.

**Figure 11:**
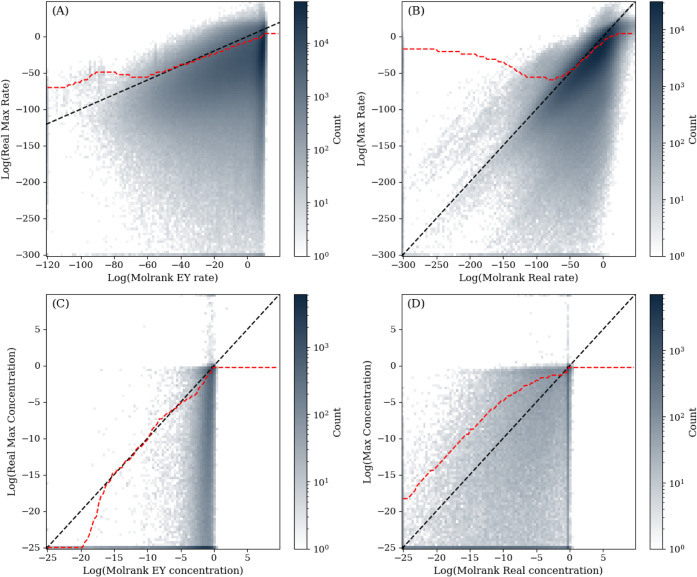
Max reaction rates and species concentration vs (A, C) MolRank score based on real kinetic rate constants and (B, D) based on Eyring–Polanyi equation. The dashed black line is *x* = *y*, meant to guide the eye, dashed red line signifies the 95th percentile of rates with MolRank score ≤ *x*.

A very similar pattern emerges when considering the maximum species concentration (Figure 11C,D). Again, EY-based MolRank provides a noticeably better bound than rate-constant-based MolRank. In fact, for species concentration, the MolRank computed from true rate constants often fails to serve as a strong bound, whereas the EY-based version maintains a consistent bounding behavior across the distribution. This further supports the notion that the EY-based formulation is applicable in practical scenarios, where only reaction energetics rather than full kinetic data are available. The correlation between approximated (EY) MolRank scores and accurate ones is presented in Figure S17. It shows that while EY scores are consistently higher than the accurate ones, the correlation is moderate, meaning that the ranking quality may deteriorate despite the better bounding qualities. Overall, the simulation results demonstrate that MolRank is a viable and practical method for bounding both maximum reaction rates and maximum species concentrations in complex networks, with somewhat increased bounding quality for species.

For published reaction networks, the availability of kinetic data makes direct evaluation possible, allowing us to test MolRank on real chemical systems (Figures S12 and S13). In contrast to the simulation results, we find that MolRank computed from the true rate constants provides a better bound on maximal species concentration and reaction rate than the EY-based version. Nonetheless, both the EY-based and rate constant-based MolRank provide decent bounds for species concentration and reaction rates in these real networks. The different trends seen in simulations likely stem from the more complex and system-specific relationship between activation energy and reaction energy in experimental data sets, compared to the simplified and more uniform mapping used in simulated networks. These findings suggest that while simulations are invaluable for systematic testing, real-network behavior can deviate because of richer underlying energetics and kinetic correlations.

#### Kinetic Validation

The kinetic accuracy of the MolRank reduction algorithm was assessed by using published combustion networks as benchmarks. In this test, reactions and species were progressively removed according to their MolRank scores until the network was fully depleted. For each intermediate reduced network, the reaction kinetics were resolved and evaluated deviations from the full reference mechanism using two metrics: (i) average reaction rate profile error and (ii) average species concentration profile error.

For reaction-based reduction (Figure 12a and b), all mechanisms exhibit a generally linear increase in kinetic error with increasing MolRank threshold values. In the ammonia and methane networks, all error metrics rise gradually to approximately 10^–3^ threshold value, at which point the entire mechanism becomes disconnected because reactions beyond this threshold are no longer reachable from the reactants. Within this range, errors start at ∼10^–12^ M and ∼10^–7^ M·s^–1^ and are no larger than 10^–2^ M and 10^4^ M·s^–1^ for concentrations and rates, respectively. These threshold values correspond to the removal of ∼13% of the reactions in the system, encouraging successive applications of the MolRank ranking for further reduction. For the hydrogen network, MolRank scores are generally higher, so the phenomenon occurs at a threshold value of 10^3^, with error ranges between 10^–6^ M·s^–1^ to 10^4^ for rates and 10^–8^ M to 10^–2^ M for concentrations.

**Figure 12:**
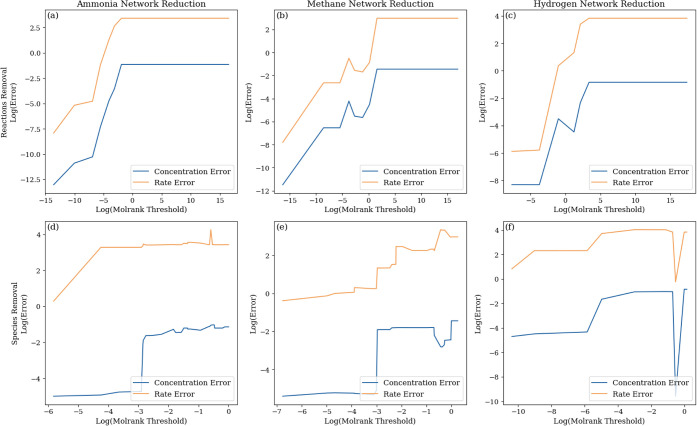
Concentration and rate log-errors vs MolRank threshold value for removal of (a–c) reactions from ammonia, methane, and hydrogen networks, (d–f) species from ammonia, methane, and hydrogen networks.

For species-based reduction (Figure 12d–f), the concentration errors show a clear step-like behavior, remaining relatively small (≈10^–5^ M) until a threshold value of ∼10^–3^ (10^–6^ for the hydrogen network), after which they increase toward 10^–2^ M. Rate errors in this regime increase more continuously, ranging from 10^0^–10^4^ M·s^–1^. This threshold corresponds to the removal of ∼20% of species and ∼25% of reactions. The higher removal rates reveal a superior performance for species ranking compared to reaction ranking. This aligns with the simulated network results that suggest better bounding of species concentrations compared to reaction rates. Importantly, both networks show similar behavior with the same MolRank thresholds, supporting its viability to various reactive systems.

While the use of Eyring–Polanyi equation-based rate constants leads to improved bounding behavior (Figures 10, 11) this does not necessarily translate into improved ranking or reduction performance. As shown in Figure S17, the correlation between EY-based and exact MolRank scores is only moderate, indicating that the detailed ordering of reaction and species importance is not reliably preserved. Furthermore, network reduction based on EY-derived scores results in larger errors in both reaction rates and species concentrations at comparable levels of reduction (Figure S18). Additional analysis of reduction error as a function of the MolRank cutoff shows similar qualitative trends to those obtained with exact rate constants, but with consistently higher errors for the same threshold values (Figure S19). These results indicate that, although approximate rate constants are compatible with the MolRank framework due to their conservative nature, their use requires more stringent (lower) cutoff values to achieve reduction quality comparable to that obtained with accurate kinetic data. A more detailed discussion of the use of approximate rate constants within the MolRank framework is provided in the Supporting Information.

#### Preservation-Based Validation

Starting from the fully enumerated network for ammonia combustion, the objective was to assess how accurately MolRank reduction can identify and preserve chemically relevant reactions and species. Accuracy was estimated by comparing the set of reactions retained after reduction to those included in the published mechanism for the ammonia combustion. Under the assumption that greater overlap with the published reactions reflects higher fidelity, the degree of conservation provides a direct measure of the reduction accuracy.

This metric is not treated as a direct measure of the chemical validity of the reduction algorithm but as a proxy for consistency with expert-curated chemistry. Due to their massive scale, no kinetic analysis or traditional path-finding algorithms are viable for these enumerated networks, and this metric provides a way to gauge the performance of MolRank for these networks as well.

A scale comparison highlights the challenge of this task. The enumerated network contains 150,292 reactions and 101 species, whereas the published benchmark includes only 470 reactions and 30 species. Of these, only 370 published reactions can be mapped to the enumerated system due to heuristic mismatches. Thus, the set of “relevant” reactions corresponds to only ∼0.2% of the total enumerated reactions, while the relevant species make up a more substantial fraction of ∼30% of the enumerated species. These proportions indicate that accurate reduction is a highly selective problem at the reaction level, whereas at the species level there is more structural overlap to exploit.

The conservation behavior is illustrated in Figure 13A, which shows the number of “important” published reactions preserved as a function of the total number of reactions at a given MolRank threshold. The analysis reveals that for the reduction of less than 30% of reactions MolRank does not discriminate between published (“important”) and enumerated (“redundant”) reactions better than random removal. The advantage of MolRank comes into play at higher thresholds (corresponding to removal of >30% of reactions), where a preference for retaining important reactions emerges. This suggests inherent limitations in applying MolRank to reaction reduction for enumerated networks.

**Figure 13:**
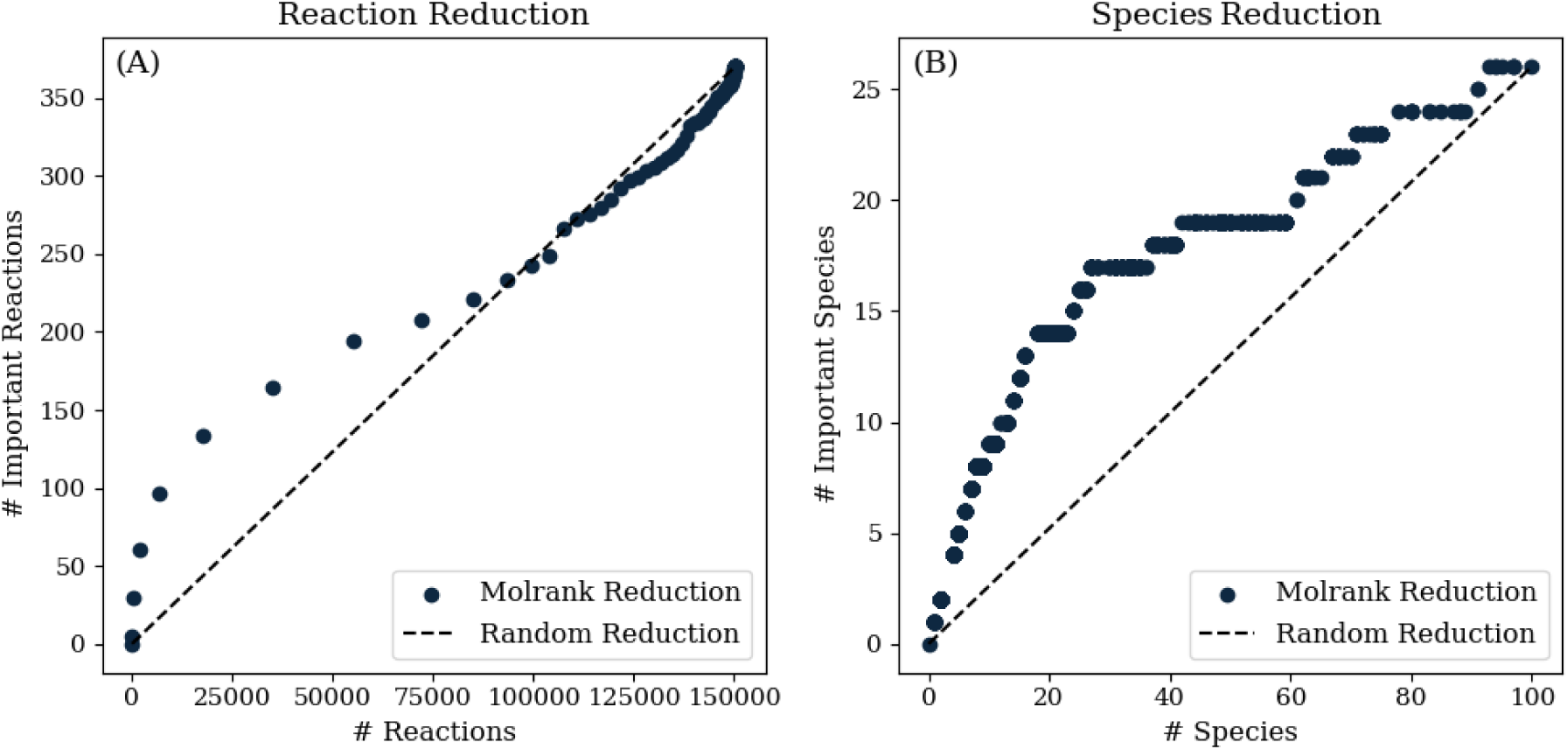
MolRank reduction performance for (A) reactions and (B) species. The plot shows the number of important objects (reactions or species) in the network vs their total number, at a given MolRank threshold. The dotted line represents random removal performance for comparison.

A stronger performance is observed in the species-level analysis as shown in Figure 13B. Even when applying modest thresholds, MolRank preferentially removes species that are absent from the published mechanism. Approximately 10% of the enumerated species, corresponding to around 10 species in the ammonia combustion case, can be eliminated without the loss of a single published species. This result is particularly significant, since the removal of a species entails the simultaneous elimination of all associated reactions, amplifying the reduction’s efficiency.

These findings suggest that while MolRank alone may not guarantee perfect separation of important and redundant reactions, it provides a powerful mechanism for the large-scale pruning of redundant species. As validated from simulation, MolRank can provide relatively accurate reductions even without full kinetic parametrization and based on only network data. In practical applications, an iterative approach may be advantageous: an initial reduction of redundant reactions and species could be followed by a recalculation of MolRank scores on the modified topology, thereby refining the accuracy of the subsequent reductions.

A commonly used tool in reaction network reduction is sensitivity analysis, which defines the importance of a reaction as the response of a selected observable to perturbations in its rate constant. As fully outlined in the Supporting Information, local sensitivities of key intermediate species’ concentrations with respect to reaction rate constants were evaluated. The resulting sensitivity coefficients, for ammonia and hydrogen networks, were compared to the corresponding MolRank scores for each reaction. Only a mild correlation (*R* ^2^ ∼ 0.3) was observed, which is not too surprising considering that the two approaches are not directly equivalent: sensitivity analysis reflects local responses to selected target observables, while MolRank reflects the overall structural and kinetic prominence of reactions within the network without respect to a specific observable.

The computational advantage of MolRank was explicitly quantified by benchmark calculations performed on a subset of 36 samples from the simulated network database by comparing MolRank score evaluation with full kinetic simulation based on ODE integration. The majority of MolRank calculations were approximately 1 order of magnitude faster for networks of comparable size, with the relative speedup increasing moderately for larger networks. Importantly, on ∼5% of tested networks solving the full kinetic ODEs was faster, which stems from the cyclic nature of the MolRank calculation. The speedup was consistent and greater than 1 order of magnitude for published networks, true for both calculations based on the accurate rate constants and the EY approximation.

## Conclusions

In this work, we have introduced two complementary methodological advances for the study and reduction of chemical reaction networks.

First, we employed a network generation model designed to produce large-scale bipartite graphs that reproduce the key structural characteristics observed in chemical reaction networks. Rather than aiming to replace detailed mechanistic models, the generator serves as a controlled environment in which the network topology can be explored independently of complete kinetic information. This capability enables the construction of reaction networks at scales that substantially exceed those of commonly available published mechanisms, thereby providing a practical test bed for evaluating reduction algorithms and network analysis methods. Comparisons with both fully enumerated and published combustion mechanisms indicate that the generated networks reproduce essential connectivity patterns while also highlighting limitations in reproducing detailed kinetic attributes such as activation energy distributions. The stronger agreement observed for real mechanisms suggests that hybrid approaches, combining chemically informed constraints with stochastic graph generation, may further improve the representativity.

Second, we introduced MolRank, a topology-based ranking algorithm, designed for network reduction. MolRank leverages graph structure to prioritize species and reactions, enabling reduction decisions to be made with *thermodynamic information only*. This method is highly scalable and capable of operating on networks far beyond the reach of traditional methods that rely on *full-kinetic simulations*. Extensive validation on both simulated and real networks confirms that MolRank can achieve meaningful network reduction with elevated performance for species over reaction reduction. Nonetheless, limitations exist: the method can misclassify reactions or species in complex topologies, and removal rates of single MolRank ranking are limited. Furthermore, the validation of MolRank on fully enumerated networks suffers from a lack of standard kinetic validation due to the size of the starting network.

These developments provide a formal framework for rigorous testing and validation of reduction strategies. By enabling comparisons across simulated and real networks, they pave the way for systematic benchmarking of advanced protocols, including machine-learning-based reduction schemes. MolRank offers a computationally efficient complement to conventional thermokinetic approaches, and the generator model provides the first pathway to testing reduction algorithms on truly large-scale networks.

Looking ahead, several directions have emerged. Expanding the generator to atmospheric, biochemical, and catalytic systems would broaden its relevance and further test the universality of the MolRank. Iterative reduction strategies, in which rankings are updated dynamically as the network evolves under pruning, could potentially improve accuracy. More broadly, these tools contribute to the automation of reaction mechanism analysis, enabling the high-throughput exploration of chemical reactivity landscapes. Overall, this study establishes both a new model for reaction network generation and a new paradigm for reaction network reduction with potential impact across combustion science, catalysis, and beyond.

## Supplementary Material



## Data Availability

The code and data used for this work are available at https://github.com/ajr15/CRNProject. More details about the implementation are found in the SI of this manuscript.

## Notes

Full details on network generation model fit, detailed ERE procedure, complete data on validation of network generation model and published networks, additional details on algorithms and implementation, full correlation plots for simulated graphs, analysis of fast equilibrium reactions in simulated networks and further analysis of MolRank algorithm (PDF)
