# Supplementary material for: Graph-Based Generation and Reduction of Complex Chemical Reaction Networks
Source: J Chem Inf Model. 2026 Jul 8;66(14):8349–60. doi: 10.1021/acs.jcim.6c01309 (PMC13417878; doi:10.1021/acs.jcim.6c01309)
Supplement: Supplementary file 1 [file ci6c01309_si_001.pdf]

SUPPORTING INFORMATION FOR: GRAPH-BASED GENERATION AND REDUCTION OF COMPLEX CHEMICAL REACTION NETWORKS

Shachar Fite[1] and Zeev Gross[1, *]

[1] Schulich Faculty of Chemistry, Technion—Israel Institute of Technology, Haifa 32000, Israel
*- Email: chr10zg@technion.ac.il

1 Contents

2 Network Generation Model 2
2.1 Prior Distribution Fitting for Network Generation Model 2
2.1.1 Specie Energy Prior 2
2.1.2 Arrhenius Parameters Priors 3
2.2 Simulated Graph Database 5
3 Elementary Reaction Enumeration 6
3.1 Enumeration Details 6
3.2 Molecular Hashing 8
3.3 Energy Calculation Details 9
4 Validation of Network Generation Model 10
5 Modified Dijkstra's Shortest Path Algorithm 11
6 Implementation Details 12
7 Properties of Published Networks 13
8 Full Correlation Plots for Simulated Graphs 15
9 Prevalence of Fast Equilibrium Reactions 15
10 Prevalence of Non-Conserving Patterns in Simulated Graphs 16
10.1 Identifying Non-Conserving Reaction Cycles 16
10.2 Prevalence in the Simulated Network Database 16
11 Further MolRank Information 18
11.1 Use of Approximated Rate Constants 18
11.2 Comparison to Sensitivity Analysis Metrics 20
12 Works Cited 21

2 Network Generation Model

2.1 Prior Distribution Fitting for Network Generation Model

As described in the Methods section of the main text, the network generation model couples a topological preferential-attachment mechanism with chemically informed priors for species thermodynamic and reaction kinetic properties. These priors provide statistically realistic property values for sampled species and reactions, ensuring that the generated chemical reaction networks (CRNs) remain consistent with known chemical behavior.

Two categories of priors are required:

- Specie energy prior: Enthalpy of formation (ΔH_f) for each species, which influences thermodynamic feasibility and equilibrium tendencies in the network.
- Reaction kinetic priors: Arrhenius parameters for each elementary reaction: the pre-exponential factor (A), temperature exponent (β), and activation energy (E_a). These govern the reaction rate coefficients and, in turn, influence the kinetic accessibility of reaction pathways.

In the following subsections, we detail the fitting procedures for these two prior distributions: (i) fitting of specie enthalpies of formation, and (ii) fitting of Arrhenius parameters from representative reaction datasets. The fitting was made on the a dataset composed from the benchmark reactions detailed in the main text. In total, 228 specie records and 1,008 reaction records (containing duplicates) were used. Each fitting step involves statistical analysis of curated reference datasets, model selection for the most appropriate probability distribution, and evaluation of the goodness-of-fit to ensure reliable sampling during network generation. Throughout all the estimations, the final prior is set for 1000K for temperature-dependent properties, as this temperature is in the relevant range of the combustion reactions in the benchmark.

2.1.1 Specie Energy Prior

The thermodynamic properties of each species are given as a temperature-dependent NASA polynomial. Figure S1 shows their distribution at different temperatures and Table S1 summarizes their statistical features. Enthalpy is the least sensitive to the temperature with average enthalpy changing by 25% between 400K and 1000K. This renders the enthalpy as a stable metric for generation. Additionally, the enthalpy distribution is drawn from a normal distribution with $p<0.05$ using the Shapiro-Wilkinson (SW) test. Thus, the prior for the enthalpy is a normal distribution can be sampled from Norm(1.21e+05, 1.98e+05) for 1000K reference temperature. Note that negative values are allowed.

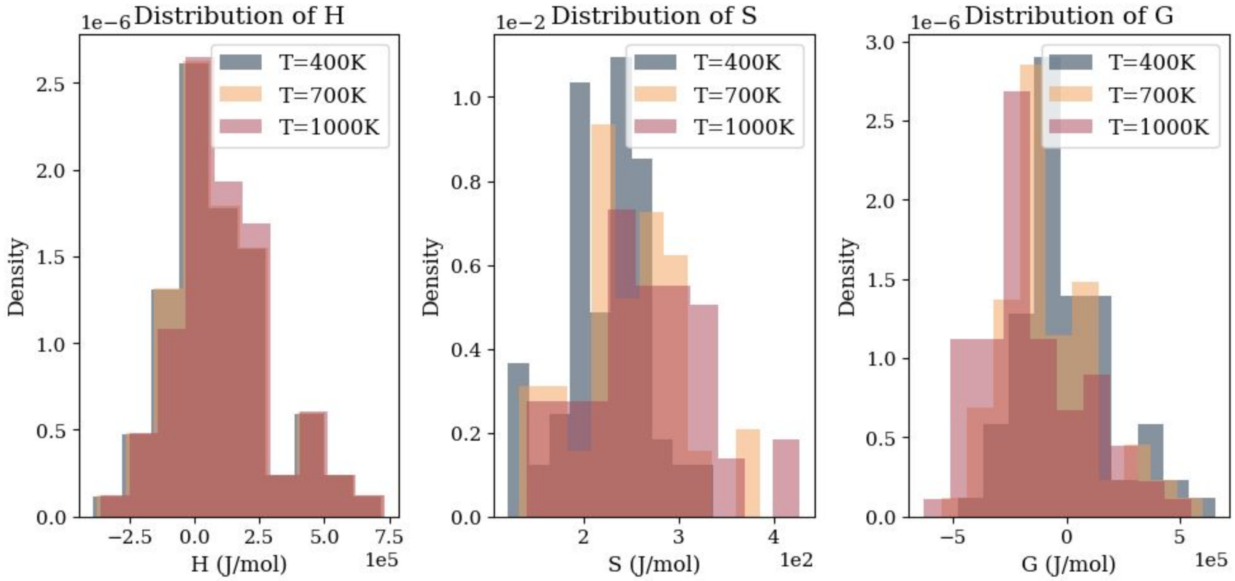

Figure S1: Distribution of specie enthalpy (H), entropy (S) and Gibbs free energy (G) for all species in database at three temperatures

Table S1: Summary statistics for each thermodynamic property. p-values are for fitting a normal distribution.

Temperature	H (J/mol)			S (J/mol)			G (J/mol)		
	**μ**	**σ**	**p**	**μ**	**σ**	**p**	**μ**	**σ**	**p**
400 K	9.07e+04	2.02e+05	0.03	2.23e+02	4.54e+01	0.21	1.63e+03	2.09e+05	0.02
700 K	1.05e+05	2.00e+05	0.03	2.49e+02	5.57e+01	0.26	-6.93e+04	2.15e+05	0.01
1000 K	1.21e+05	1.98e+05	0.04	2.68e+02	6.49e+01	0.16	-1.47e+05	2.24e+05	0.01

2.1.2 Arrhenius Parameters Priors

The Arrhenius equation consists of three independent parameters for calculating the reaction rate constant at different temperatures. From the benchmark, 504 forward reactions had direct information on their parameters, and the other reversed 504 reaction had implicit information via the forward parameters. The parameters of the reversed reaction can be estimated using the equilibrium constant of the reaction, giving

$$E_a^{(r)} = \Delta H^{(r)} + E_a^{(f)},\ A^{(r)} = A^{(f)} \exp \frac{\Delta S^{(r)}}{R\Delta n^{(r)}},\ \beta^{(r)} = \beta^{(f)} + \Delta n^{(r)}$$

Where the (r) and (f) superscripts indicate reversed or forward reaction, ΔH is the reaction's enthalpy, ΔS is its entropy and Δn is the change in gas moles in the reaction. The reversed reaction parameters are estimated at 1000K temperature. The relationship between the activation energy and the reaction entropy is given in Figure S2. It is clear that for $\Delta H > 0$ there is some scaling relation between the enthalpy and the activation energy, while for $\Delta H < 0$ there is a noisy relation.

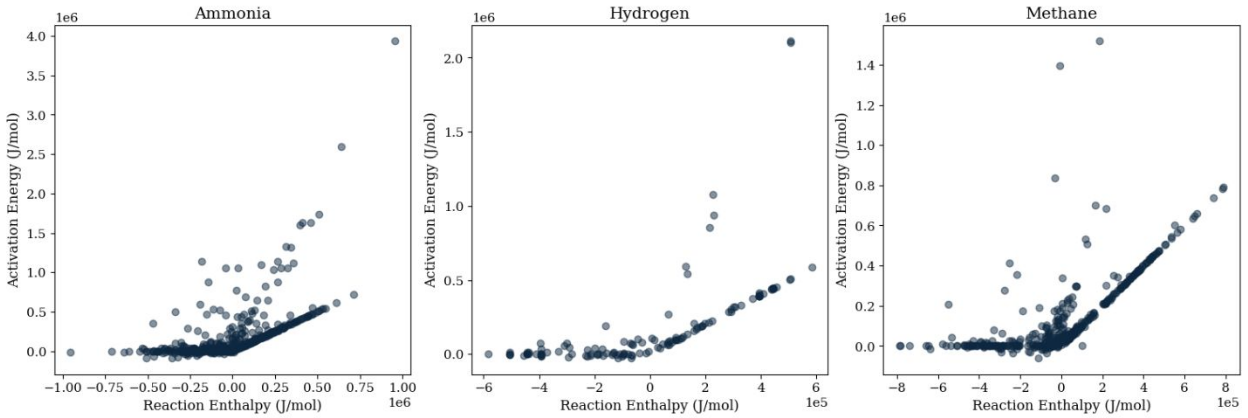

Figure S2: Relationship between E_a and ΔH_r for the benchmark reactions

The distributions of the pre-exponential factor and temperature exponent together with activation energies where $\Delta H < 0$ are shown in Figure S3 and statistics are summarized in Table S2. Plots show quite good correlation with normal distribution for $\log A$,$\log E_a$ and β suggesting a log-normal distribution for A and E_a and normal distribution for β. The SW test values for all properties are below 0.1, suggesting a good fit. For the activation energy the fit is somewhat lower, but for a lack of better model, a log-normal distribution is selected.

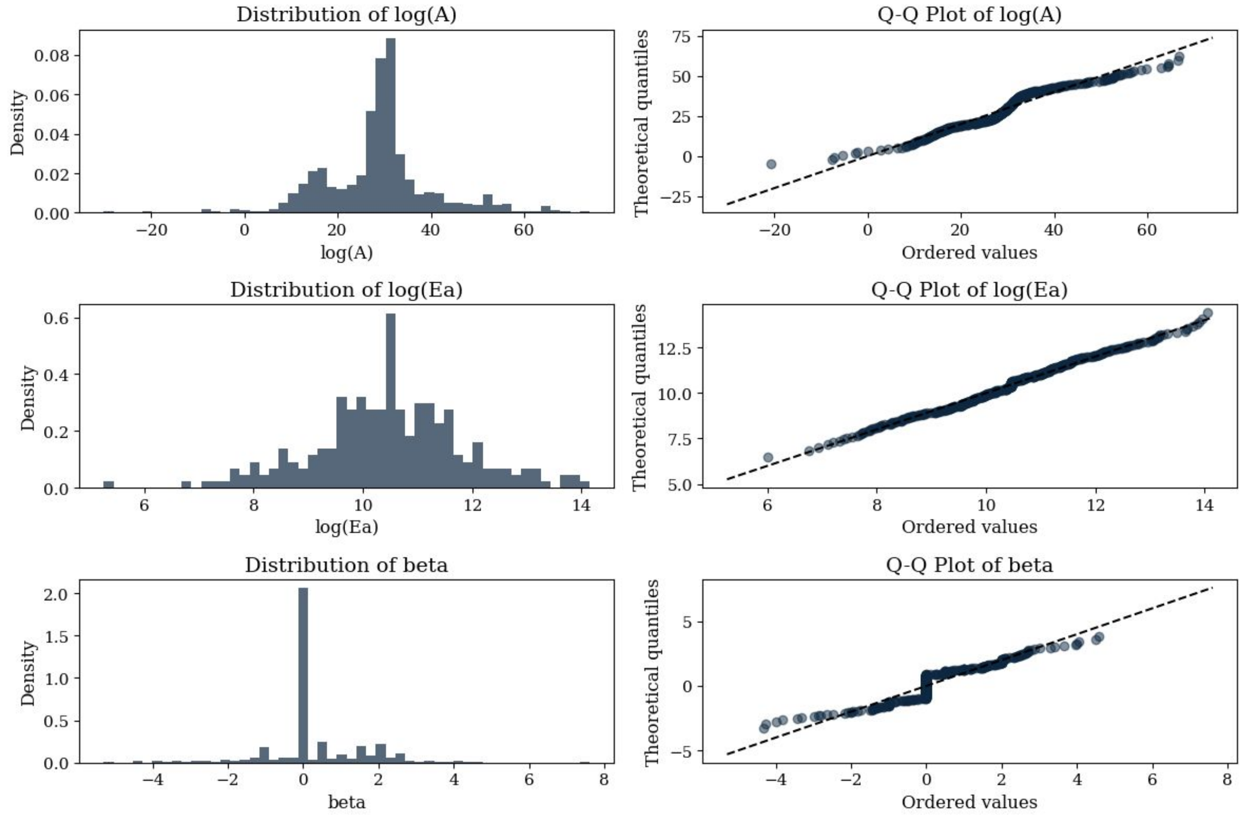

Figure S3: Distributions of Arrhenius parameters, along with Q-Q plots versus normal distribution quantiles.

Table S2: Summary statistics and distribution parameters for Arrhenius parameters

Property	μ	σ	N	p-value
$\log(A)$	28.74	11.64	1008	2.1e-20
$\log(E_a)$ (J/mol)	10.46	1.37	247	7.1e-02
β	0.32	1.23	1008	3.0e-30

Fitting the activation energy where $\Delta H > 0$ requires fitting a "scaling relation" between ΔH and E_a. Figure S4 depicts the fit performance and fit error. The scaling relation suffers from a switching linear relationship between the two properties, leading to non-normal error distribution. To keep the model simple, without adding too specific distribution parameters, a normal distribution for errors was taken despite its inaccuracies. The parameters of the prior are: slope 1.26 (unitless), intercept 3.35e+04 (J/mol), error mean 0, error std 2.66e+05 (J/mol). These parameters are used for sampling reaction activation energy when $\Delta H > 0$.

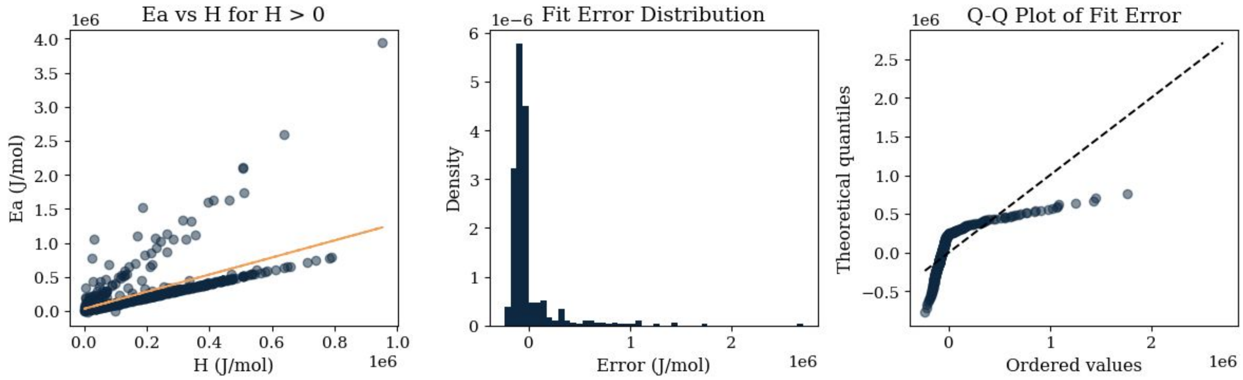

Figure S4: Scaling relation fit between reaction enthalpy (H) to activation energy (Ea) along with fit error data. Total of 499 reactions where used for fitting.

2.2 Simulated Graph Database

To enable systematic investigation of the reaction network properties and MolRank algorithm performance, a database of simulated chemical reaction networks was constructed. Graphs were sampled from a two-dimensional grid defined by the number of species and the number of reactions. The number of species was varied between 25 and 75 (20 evenly spaced points), while the number of reactions was varied between 750 and 2500 (33 evenly spaced points). For each grid point, 10 independent realizations were attempted.

For every graph generated, two species were randomly selected as origin nodes, and graph connectivity was verified. Subsequently, a kinetic simulation was performed to ensure physical plausibility. Concentration profiles were required to remain strictly positive for all species, while maximum reaction rates were validated to exceed the threshold defined for steady-state behavior. Kinetic simulations were carried out using the scipy BDF solver, with absolute and relative tolerances of 1×10^{-25} and 1×10^{-3}, respectively. Time integration proceeded until the maximum rate of change of species concentration fell below 1×10^{-6}, which was taken as the steady-state criterion.

Only networks that satisfied all connectivity and kinetic validation requirements were retained. For each trial, up to 30 attempts were performed to obtain a valid graph. Using this approach, a total of 2,613 networks were successfully generated.

The distribution of network sizes in the simulated database is summarized in Figure S5A and B. The histogram of species counts (Figure S5A) indicates that networks with lower numbers of species are more readily generated and therefore more prevalent in the database. The average number of species across the dataset is 43. In contrast, the distribution of reactions (Figure S5B) appears more uniform, suggesting that the number of reactions is less restrictive than the number of species for successful generation. The average number of reactions across the dataset is 1673.

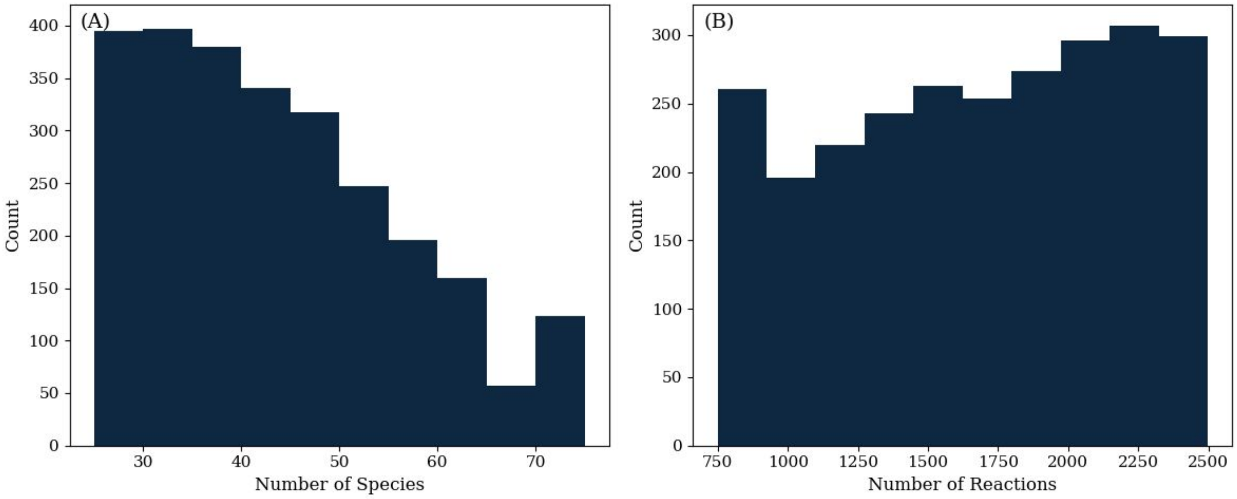

Figure S5: Distribution of (A) number of species and (B) number of reactions in simulated network database.

3 Elementary Reaction Enumeration

3.1 Enumeration Details

The results in this study are based on CRNs enumerated by TorinaNet, an in-house CRN analysis and ERE software. TorinaNet's ERE is based on the atom connectivity (AC) matrix scheme (graph-based), whose scheme is presented in detail elsewhere [1], [2] while here only a brief review is provided. An AC matrix is a symmetric matrix representing a molecular system, where its diagonal elements are equal to the atomic numbers of the atoms, and the off-diagonal elements are binary for bonding between two elements. It can actually represent more than one molecule, as a block matrix of single molecules' AC matrices. A reaction is represented as a conversion matrix (CM) transforming one AC matrix (possibly representing a few molecules) to another. The expression of a chemical reaction is demonstrated in Figure S6. The CMs can be systematically generated given an AC matrix, thus covering all possible reactions in a system.

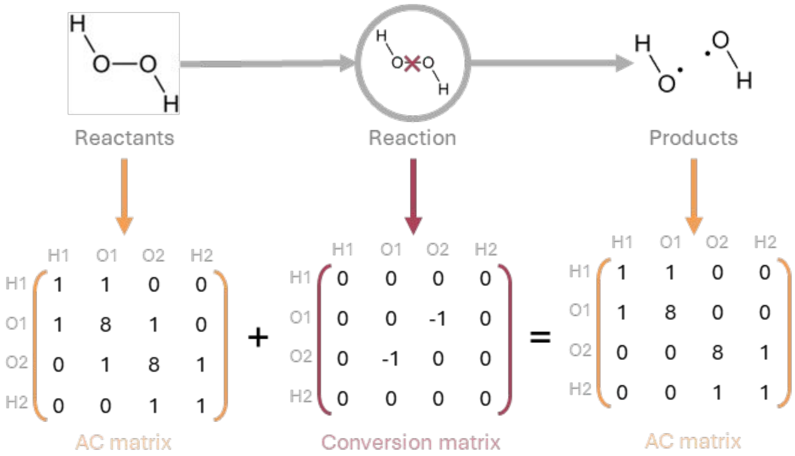

Figure S6: Hydrogen peroxide decomposition reaction expressed in terms of AC and conversion matrices. The reaction is expressed in terms of matrix addition.

A mutli-molecular AC matrix is the input to the algorithm, and all reactions between the initial molecules or any intermediate species are enumerated [2], [3], [4]. This method is fixing the total number of atoms; and are thus not well suited for reaction systems where the products or intermediates are larger than the reactants, as commonly happens in combustion or atmospheric reactions. Therefore, the enumeration mechanism in TorinaNet is slightly modified, and builds different AC matrices by combining species in the graph during enumeration. The procedure in each enumeration cycle is as follows:

0. Initialization: put the reactants in the graph. Define them as new species.
1. Single-specie enumeration: add all the single-specie reactions of new species in the graph
2. New-new enumeration: add all the two-specie reactions involving new species in the graph. Selection is *with* repetition.
3. New-old enumeration: add all the two-specie reactions involving new species in the graph with old species in the graph. Selection *without* repetition.
4. Reset species: set all the added species in the graph as new, all the rest of the species are old.

Each multi-molecular AC enumeration can be done in parallel, making the algorithm highly scalable. To ensure efficient and correct ERE, the reaction addition depicted is done via decomposition of multi-molecular product AC matrix to single-molecule AC matrices. Each molecule is checked for uniqueness (*vide infra*) and added to the graph; this is crucial to ensure proper assignment of reactions to species. Reactions are also checked for uniqueness, based on the reactants and products, and added to the graph.

The amount of uniqueness checks of species and reactions is immense in a typical ERE calculation and if not done efficiently, it can dramatically slow down the computation time. This problem of uniqueness checks is addressed by molecular hashing in various ERE systems [2], [3], [5]. In TorinaNet, the hashing is done via a molecular fingerprint calculation, as implemented in RDKit [6]. Individual molecular hash functions often lead to non-unique hash values for large enough collection of molecules (see section 3.2 below). A cuckoo hash algorithm [7] was implemented in TorinaNet to tackle incomplete hash functions.

To avoid steep scaling and keep the network accurate, multiple filters are used to remove unnecessary species and reactions. The basic filters can be divided into three main groups: conversion matrix filters, AC matrix filters and thermodynamic filters. Conversion matrix filters ensure that only valid transformations are being used and avoid exploration in unproductive directions. These filters mostly ensure that the right amount of bonds is formed or broken: specifically, in this work all reactions can at most break 2 bonds and

form 2 bonds – the so-called 2b2f rule [3], [4]. AC matrix filters are applied to the species in the network, specifically to the compound product AC matrix during enumeration. The filters' goal is twofold: (1) ensuring all reaction steps in the network are elementary and (2) controlling the scaling of the enumeration by including only chemically-relevant species and making it computationally feasible. The filters used in this work are:

- Proper valence: atoms cannot have more bonds than their proper valence values, for nitrogen there can't be more than 3 bonds, for oxygen 2 and so on.
- Max two products per reaction: only reactions that create 2 or less products are allowed
- No rings: the species are not allowed to have any rings, as they are highly unlikely to occur in our benchmark system.
- Size limit: species can have at most 2 oxygen and 2 nitrogen atoms. Hydrogen is not limited.

The last group of filters are thermodynamic: based on *ab-initio* energy calculation of each specie. The specie geometry can be readily and reliably generated from its AC matrix [1], this geometry is the starting geometry for quantum calculation (see section 3.3 below). Using calculated specie energies, reaction energies can be estimated. In most ERE systems, a simple threshold is applied to filter out thermodynamically infeasible reactions. In this work no threshold was used.

3.2 Molecular Hashing

Molecular hashing refers to the process of converting a molecular structure into a unique integer or string identifier. In principle, a perfect hashing scheme reduces the problem of molecular identity verification from a graph isomorphism comparison—computationally expensive in large systems—to a simple integer comparison. Such a transformation is of immense importance to the performance of any Elementary Reaction Enumeration (ERE) framework, particularly those relying on graph-based methods where the number of generated species can easily reach into the tens or hundreds.

The requirement for hashing arises from the need to recognize molecular equivalence. Two molecules that are structurally identical must yield the same hash value. Standard line notations such as SMILES are insufficient in this regard, since atom ordering can yield distinct strings for identical molecules, making them unreliable as direct hash functions. To address this limitation, several alternative strategies have been developed. Among the most widely used are molecular fingerprints — bit string representations encoding structural motifs—and eigenvalue spectra derived from the adjacency (AC) matrix of a species.

The performance of different hashing strategies was systematically evaluated by analyzing the number of duplicate hash values produced for large collections of molecules (Figure S7). Test species were generated by graph enumeration starting from simple feedstocks such as ammonia, oxygen, and methane. Figure S1 reports the average number of duplicate hashes obtained as a function of the number of species in the collection, calculated over 10 independent sampling repetitions. The results reveal that all fingerprint-based hash functions exhibit imperfections: distinct species can occasionally collapse to the same hash value. Although not seen in the figure, eigenvalue-based can suffer from degeneracy in larger datasets.

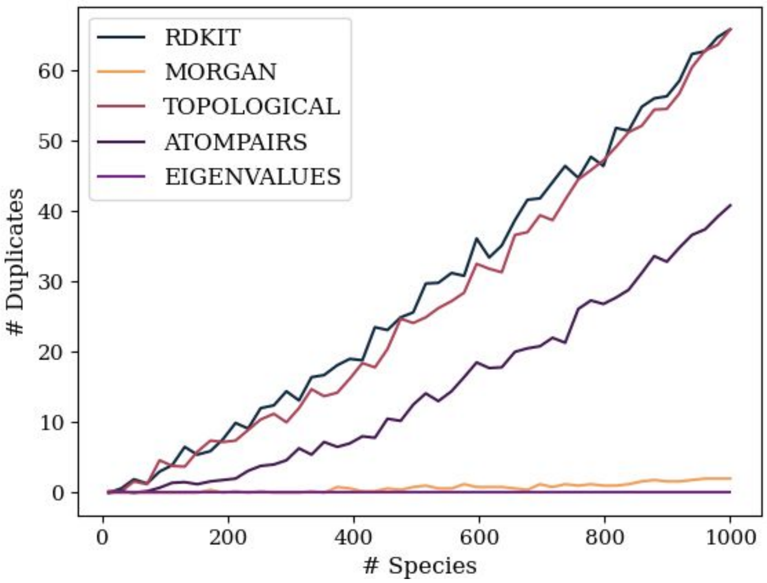

Figure S7: Number of duplicate hash values vs number of species in collection. Species generated by sampling reaction graph of nitrogen, oxygen and methane.

Because imperfect hashing cannot be fully avoided in practice, robust collision-handling schemes must be employed. In the present work, a Cuckoo hashing algorithm was utilized, which retains the performance advantages of hashing while resolving conflicts that arise from non-unique mappings. This strategy ensures reliable molecular indexing even in extremely large-scale enumerations, thereby enabling the tractability of graph-based ERE simulations.

3.3 Energy Calculation Details

Each specie in the graph underwent a geometry optimization to estimate its ground state energy. The initial-guess geometry was generated using openbabel from the AC matrix followed by a molecular-mechanics structure optimization. The resulting structure was passed to an UHF-def2-SVP geometry optimization in gas phase. Ground state spin multiplicity was estimated using openbabel, and all specie charges are zero. All computations were done using the ORCA 5 program.

Figure S8 shows the correlation between calculated energies and reported reaction energies for 451 reactions in the published networks. The average absolute error is about 2.09 eV for reaction energies, which is a reasonable error for *ab-initio* calculation at this level of theory, and is deemed sufficient for the purpose of this research.

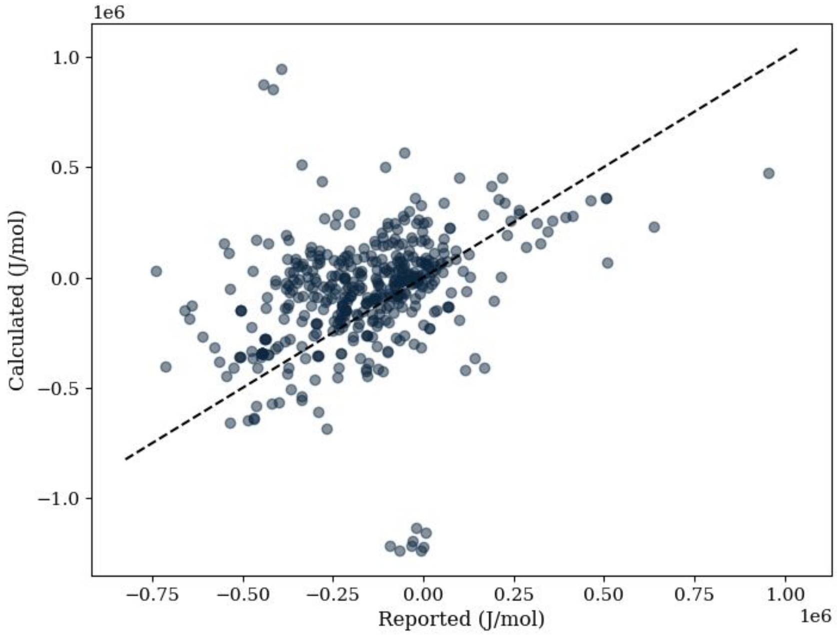

Figure S8: Energy fit plot for reported reaction energies and calculated energies, based on 451 reactions from published mechanisms.

4 Validation of Network Generation Model

Table S3: P-values calculated by a Kolmogorov-Smirnov (KS) test for degree distribution fits. In green are cells where the fit is significant (95% confidence)

Reaction	Degree	Preferential Attachment		Erdos-Renyi	
		statistic	P	statistic	P
Hydrogen	In	0.23	0.53	0.40	0.05
Hydrogen	Out	0.15	0.94	0.38	0.06
Hydrogen	Total	0.31	0.21	0.48	0.01
Ammonia	In	0.17	0.42	0.33	0.01
Ammonia	Out	0.19	0.26	0.33	0.01
Ammonia	Total	0.10	0.93	0.39	0.00
Methane	In	0.21	0.15	0.48	0.00
Methane	Out	0.14	0.57	0.47	0.00
Methane	Total	0.24	0.06	0.56	0.00

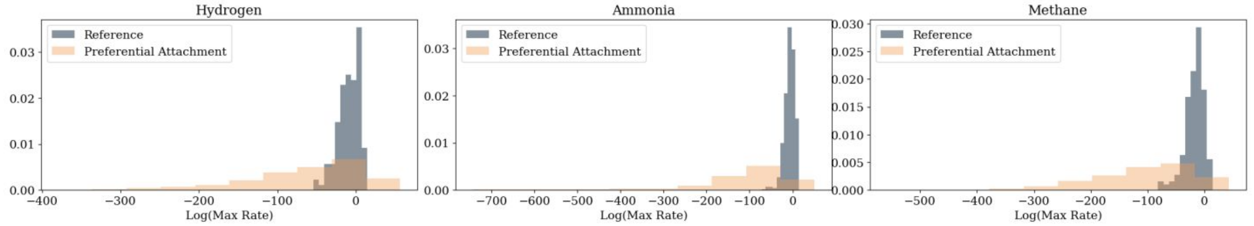

Figure S9: Distribution of log(max rate) of reference published network reactions and generated ones

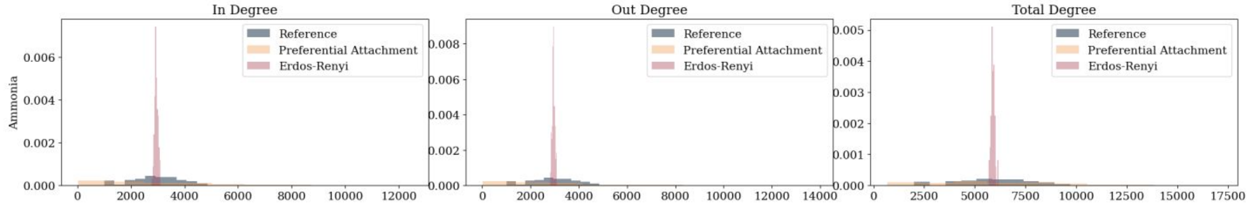

Figure S10: Degree distribution of enumerated ammonia network compared with preferential attachment and Erdos-Renyi degree distributions.

5 Modified Dijkstra's Shortest Path Algorithm

Shortest-path distances were computed between each species and the primary reactants of the network. This was achieved through a modification of the classical Dijkstra shortest path algorithm. In contrast to standard Dijkstra's algorithm, which considers only nodes and edges, the chemical network is bipartite: one node class corresponds to species, and the other to reactions. The path length is therefore defined not only by species adjacency, but also by the sequence of reactions required to produce a given species from the source reactants.

The algorithm steps are as follows:

1. Initialization: All species are assigned an initial distance of infinity. Source species (initial reactants of the network) are assigned distance zero.
2. Selection of next species: At each iteration, the unvisited species with the shortest current distance from the source is selected, as in the classical algorithm.
3. Reaction-based distance updates: Instead of updating directly via graph edges, the algorithm checks reactions for which all reactant species have already been visited. Once a reaction is activated, its products can be assigned a new distance. The distance estimate is defined as the maximum distance of the reactant species plus a weight given by a property function. Different weight functions can be used for the reactions (energy, count, etc.) for different path calculations.
4. Updating product distances: For each product of the activated reaction, the algorithm checks whether the new distance estimate is smaller than its currently recorded value. If so, the distance and the producing reaction are updated.
5. Termination: The loop continues until all species have been visited. The result is a distance mapping for each species, along with the reaction pathway through which it is reached.

A pseudo-code for the algorithm is

```
Input:
reaction graph = G
```

```
source species set = S0
reaction property function = prop_func

Initialize:
  for each species s in G:
      distance[s] ← ∞
      rxn[s] ← None
      visited[s] ← False
  for each s in S0:
      distance[s] ← 0

While ∃ unvisited species:
    u ← unvisited species with minimum distance[u]
    visited[u] ← True

    For each reaction r ∈ successors(u):
        if all reactants of r are visited:
            d_est ← max(distance[reactants of r]) + prop_func(r)
            For each product p of r:
                if d_est < distance[p]:
                    distance[p] ← d_est
                    rxn[p] ← r

Output: table {species: (distance[species], rxn[species])}
```

The algorithm explicitly enforces reaction feasibility by requiring that all reactants of a given reaction must already be accessible before its products can be considered. This mimics chemical causality: products cannot be formed before all necessary reactants are present. The distance is calculated as the maximum of the reactant distances rather than their sum, reflecting the fact that the limiting reactant in a reaction determines the earliest possible time at which the products can appear.

6 Implementation Details

The entire code for this project was written in the Python programming language. Generation and analysis of CRNs was done by the TorinaNet package available at https://github.com/ajr15/TorinaNet/tree/v1.0, automatic quantum calculations and IO handling was done by the TorinaX package available at https://github.com/ajr15/TorinaX. Statistical tests were performed using the scipy python package [8]. All calculations were based on the ORCA 5 software [9], full details are in section 3.2 in the SI. Kinetic simulations were done using a custom implementation in TorinaNet based on the scipy package with full details in section 2.2 in the SI. The code used to make this paper is available at https://github.com/ajr15/CRNProject.

7 Properties of Published Networks

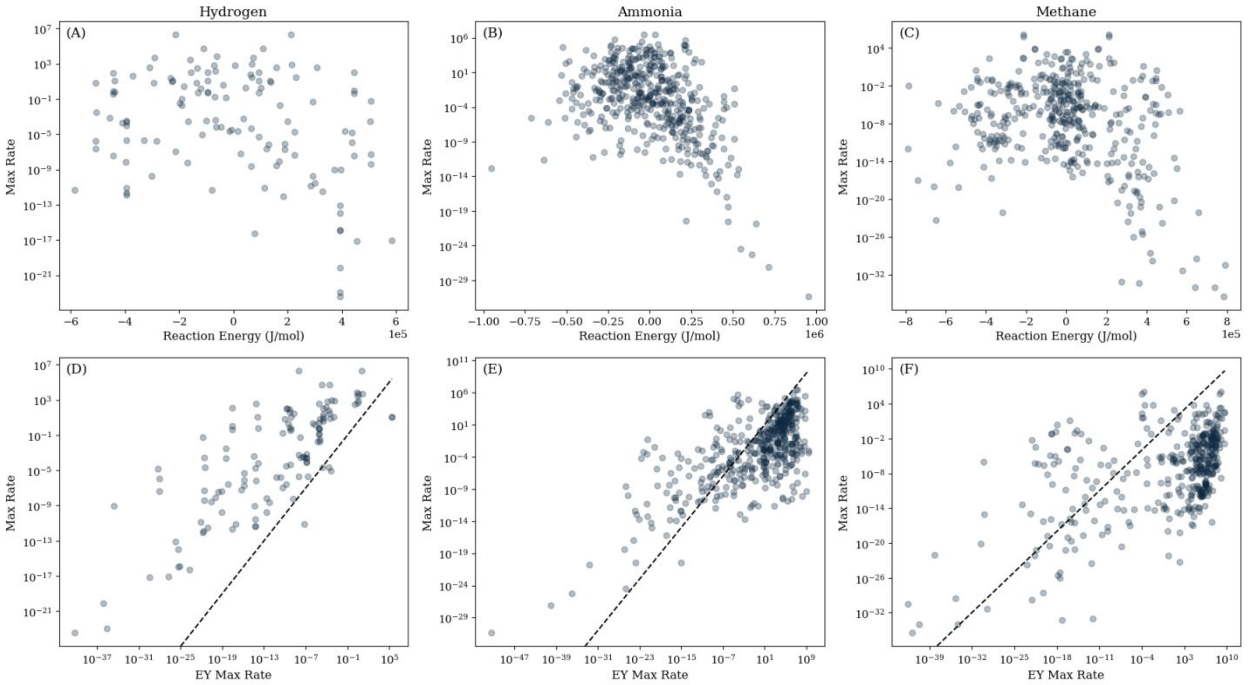

Figure S11: Correlation between energy and max reaction rate (A-C) and between max rate calculated by EY approximation to real max rate (D-F) for all 3 published networks

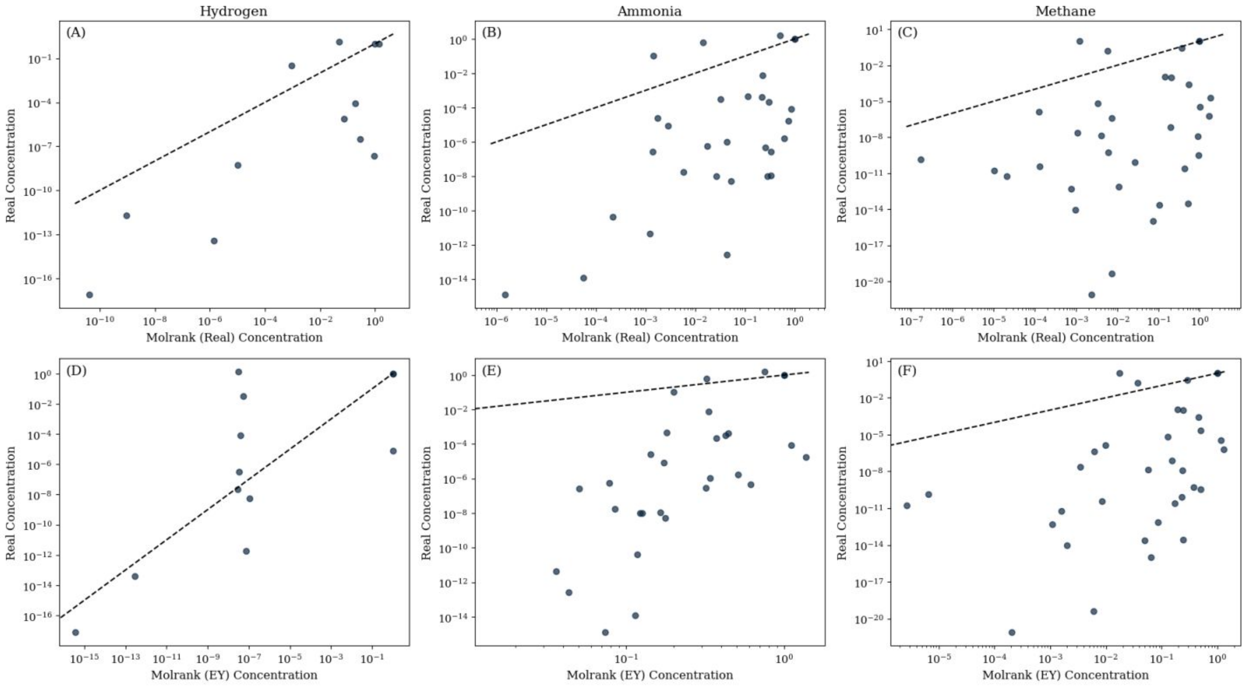

Figure S12: Correlation between Molrank max concentration estimates based on real rate constants and max specie concentration (A-C) and between Molrank max concentration estimates based on EY rate constants and max specie concentration (D-F) for all 3 published networks

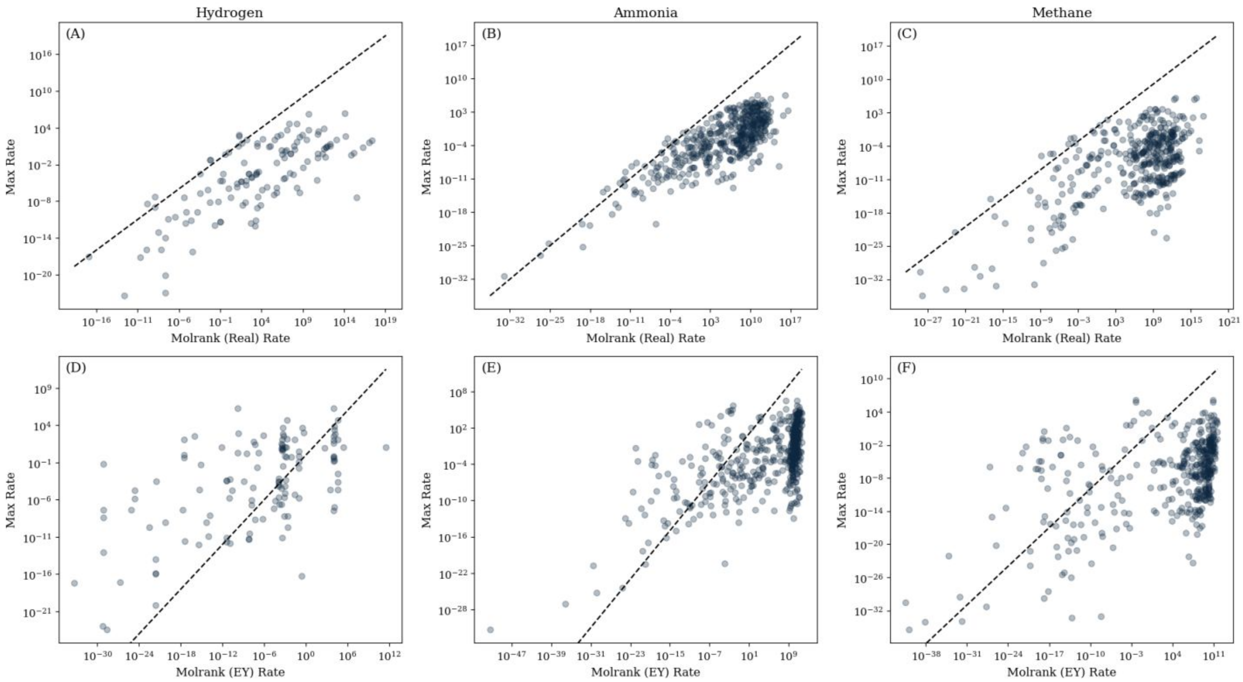

Figure S13: Correlation between Molrank max rate estimates based on real rate constants and max reaction rate (A-C) and between Molrank max rate estimates based on EY rate constants and max reaction rate (D-F) for all 3 published networks

8 Full Correlation Plots for Simulated Graphs

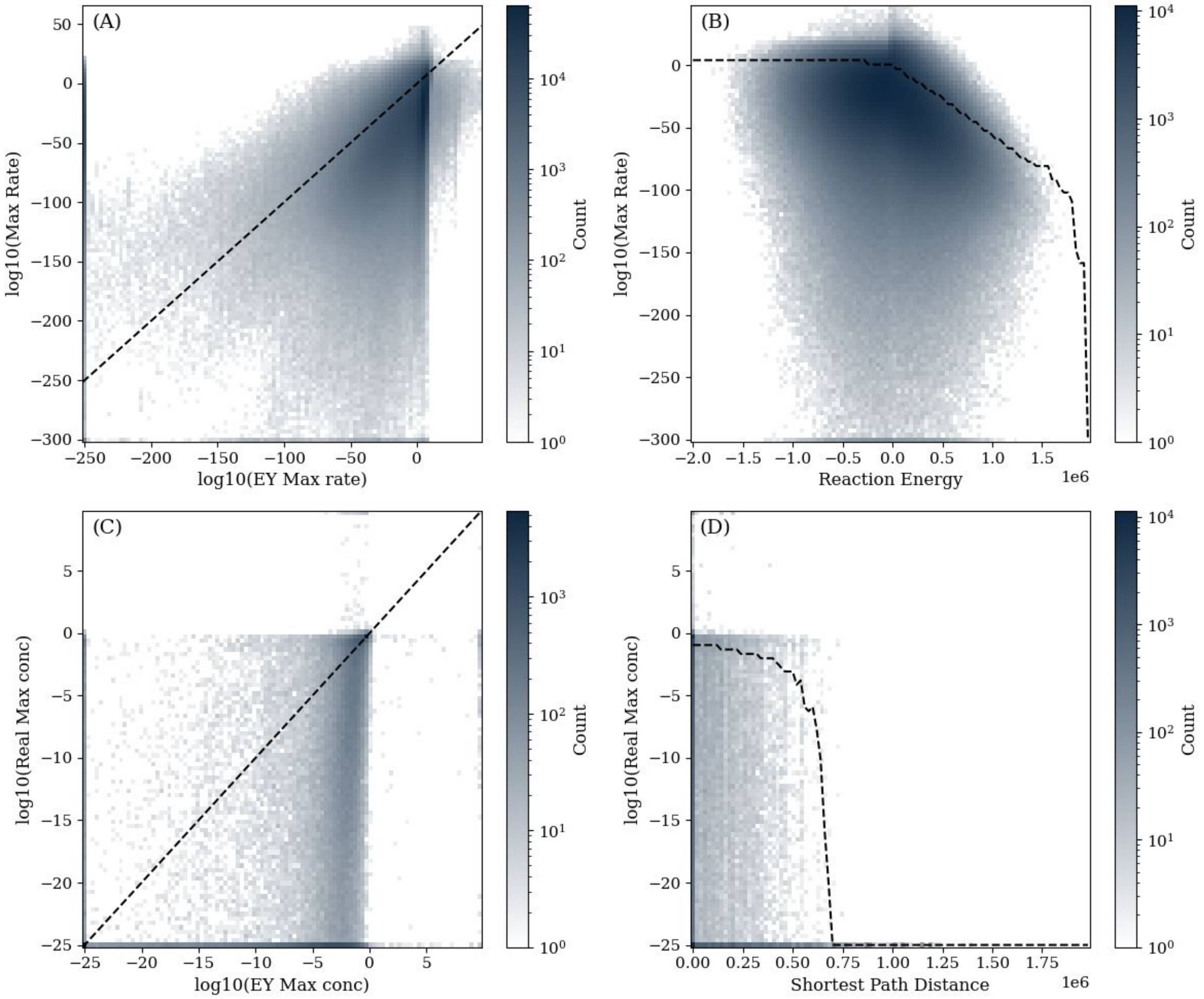

Figure S14: Correlation of (A) maximal reaction rate by EY approximation and (B) reaction energy with maximal reaction rate. Dotted lines are (A) x=y and (B) top 5% largest values to guide the reader for trends. (C) and (D) are correlations of maximal species rate by EY approximation and shortest energy path to maximal species rate respectively. Dotted lines are again (C) x=y and (D) top 5% largest values.

9 Prevalence of Fast Equilibrium Reactions

As mentioned in the main text, the MolRank approximation is expected to fail on network topologies with species that exhibit a “fast equilibrium”. This is because such species might have a small flux in a forward reaction of an equilibrium, while MolRank will predict large flux for larger rate constant reactions. To quantify this topology in both simulated and published mechanisms we counted the number of species in which (1) the forward rate constant of a reversible pair accounts for more than 50% of the total outgoing rate constants, and (2) the corresponding equilibrium rate does not exceed 10^3, indicating strong presence of this specie in equilibrium. The test revealed that no simulated network had this topology, as explicitly reversible reactions are rare by construction. In published networks, methane network had 3 species,

ammonia network had 5 species and hydron network had 3 species. This indicates that while the limitation is relevant in general, it is not dominant for the combustion chemistries examined here.

10 Prevalence of Non-Conserving Patterns in Simulated Graphs

As discussed in the main text, the generated reaction graphs are constructed from topological sampling rules rather than chemically assigned species identities, they do not explicitly enforce energy and mass conservation laws [10]. As a result, some sampled networks may contain nonphysical reactions or reaction cycles, that violate these constraints. Following the criteria detailed by Muller in [10], it was found that the simulated networks in the database preserve energy (thermodynamically sound), but do not conserve mass. As mass conservation is defined as the ability to assign "masses" to the species in the network, it does not explicitly identify the non-conserving motifs. Such an identification is unfortunately not easily achievable for networks without explicit mass and elemental composition assignment.

To complete the discussion on the implications of non-conserving networks, an approximate quantification of non-conserving motifs is presented and analyzed based on the simulated graph database.

10.1 Identifying Non-Conserving Reaction Cycles

As demonstrated in [10], the non-conserving nature of a network is completely determined by its stochiometric matrix, $\boldsymbol{S}$. In this matrix, rows correspond to species and columns correspond to reactions. Each element S_{ij} represents the net stoichiometric change of species i in reaction j: negative values indicate consumption, positive values indicate production, and zero indicates that the species does not participate in the reaction. Thus, the matrix provides a compact representation of the connectivity and net material flow of the reaction network.

A non-conserving reaction cycle was defined as a positive, nonzero vector c satisfying $\boldsymbol{S}c \geq 0$ where the inequality is interpreted elementwise and additionally $\boldsymbol{S}c \cdot \vec{1} > 0$, where $\vec{1}$ is a vector with all entries equal to 1, the length of c. Such a vector represents a combination of reactions that results in a net positive production of at least one participating species. In topological terms, this corresponds to a cycle that creates new moles within the network (cornucopia).

Detection of non-conserving cycles was formulated as a linear programming (LP) problem and solved iteratively as follows:

1. Construct the stoichiometric matrix S for the network
2. Solve the LP problem to identify a nonzero vector x such that: $\boldsymbol{S}c \geq 0$ and $\boldsymbol{S}c \cdot \vec{1} > 0$
3. Store the identified cycle vector as a detected non-conserving cycle.
4. Add orthogonality constraints to exclude previously identified solutions: $\boldsymbol{W}c = 0$ where the rows of W contain all previously detected cycle vectors.

Repeat steps 2–4 until no additional feasible solutions are found. In the solution we allow non-integer values for the cycle vector to save computation time. This procedure yields a set of orthogonal non-conserving cycles. Two limitations of this approach should be noted: (1) The criterion probes only molar non-conservation and does not detect violations of elemental or mass balance and (2) The method provides only a partial characterization of possible violating motifs and does not guarantee exhaustive enumeration.

10.2 Prevalence in the Simulated Network Database

Figure S15A shows the distribution of the number of detected non-conserving cycles across the simulated network database. In general, each reaction participated in at most one such cycle, and the total number of

cycles per network was low, corresponding on average to approximately 1% of the total number of reactions (Figure S15B). This indicates that non-conserving motifs are relatively sparse in the sampled graph ensemble.

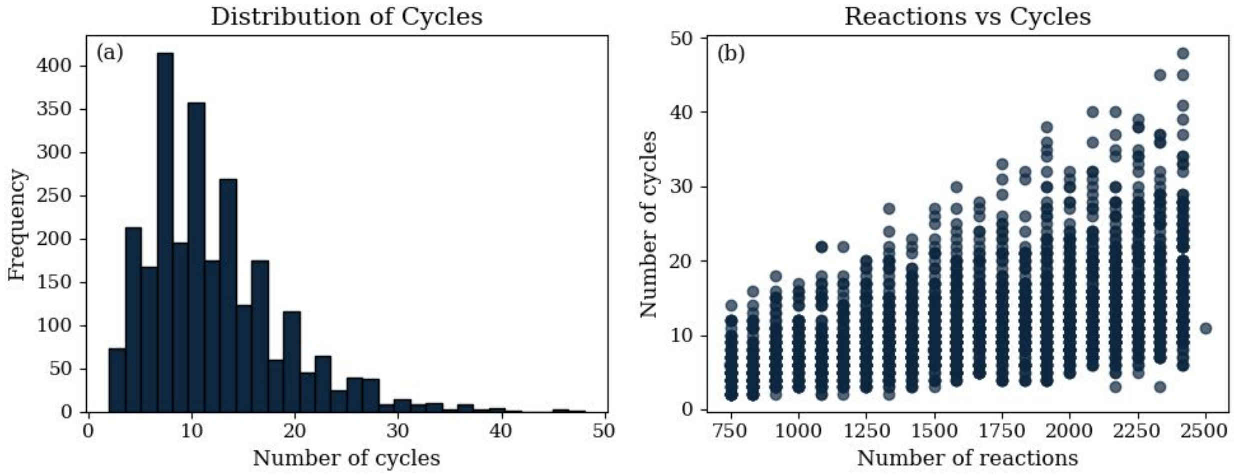

Figure S15: (a) distribution of number of non-conserving cycles, and (b) number of cycles in network vs. the number of reactions in the network.

Following the cycle identification, a measure for how much a single reaction is "non-conserving" can be defined as the number of cycles a reaction participates in. In practice, this score is only 0 or 1 for all reactions in the simulated network database. Correlating this participation score with the reaction redundancy metric, reveals that on average, reactions with higher redundancy scores (more relevant) also tend to participate in non-conserving cycles (Figure S16). This can be explained by the fact that relevant reactions will be central in the graph (topologically) and thus will have high likelihood of being included in such cycles.

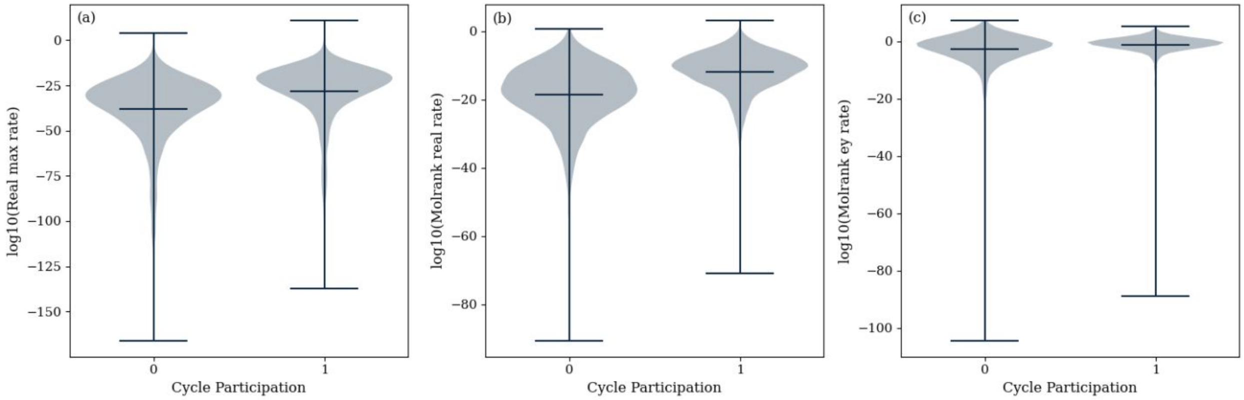

Figure S16: Violin plot of different reaction redundancy scores vs. number of non-conserving cycles a reaction participates in for (a) max rate from full kinetic computation, (b) MolRank score based on accurate rate constants and (c) MolRank based on EY approximated constants.

Overall, these results indicate that, within the limits of the present proxy definition, non-conserving motifs are relatively uncommon in the simulated graphs and tend to be retained rather than preferentially removed by the ranking procedures considered here. However, this should not be interpreted as proof that reduced networks do not preserve conservation laws, since no explicit post-reduction conservation analysis was performed and the present metric captures only one restricted form of non-conservation.

11 Further MolRank Information

11.1 Use of Approximated Rate Constants

As discussed in the main text, MolRank scores computed using Eyring–Polanyi equation (EY)-based rate constants provide consistently stronger upper bounds for both species concentrations and reaction rates compared to those obtained using exact kinetic data (Figure 11). This behavior originates from the conservative nature of the EY approximation, which systematically overestimates rate constants and, consequently, propagates into higher MolRank scores (Figure 10). This confirms that the use of approximate rate constants is theoretically compatible with the MolRank framework, as the bounding property required by its derivation is preserved.

One still has to keep in mind that this improved bounding behavior does not imply improved ranking performance. Figure S17 shows the correlation between MolRank scores obtained using EY-based and exact rate constants. While the EY-based MolRank scores are consistently higher than the real ones, the correlation is only moderate, indicating that the detailed ordering of reaction and species importance is not reliably preserved. This has direct implications for the practical application of MolRank in network reduction.

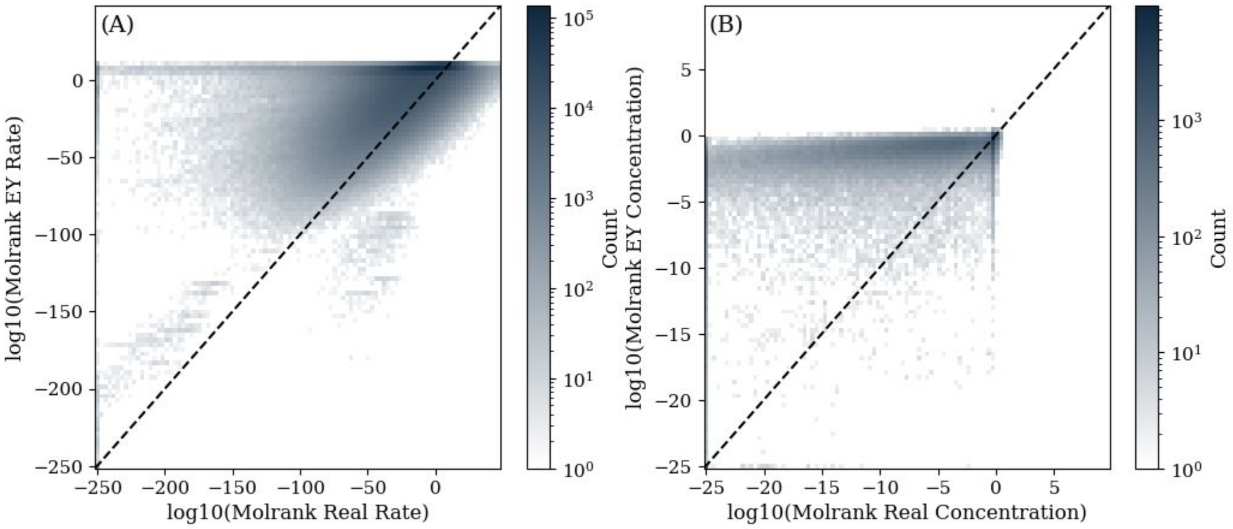

Figure S17: correlation between MolRank scores based on real and approximated rate constants for (A) reaction rates and (B) specie concentrations. The correlation is measured on data from the simulated network database.

Figure S18 shows the reduction errors, as defined in the kinetic validation section of MolRank in the main text, as a function of the reduction factor (i.e., the number of reactions or species in the reduced graph divided by the original number). For species ranking, EY-based reduction yields similar (but higher) errors compared to MolRank based on accurate rate constants. For reaction ranking, the errors associated with EY-based reduction are higher.

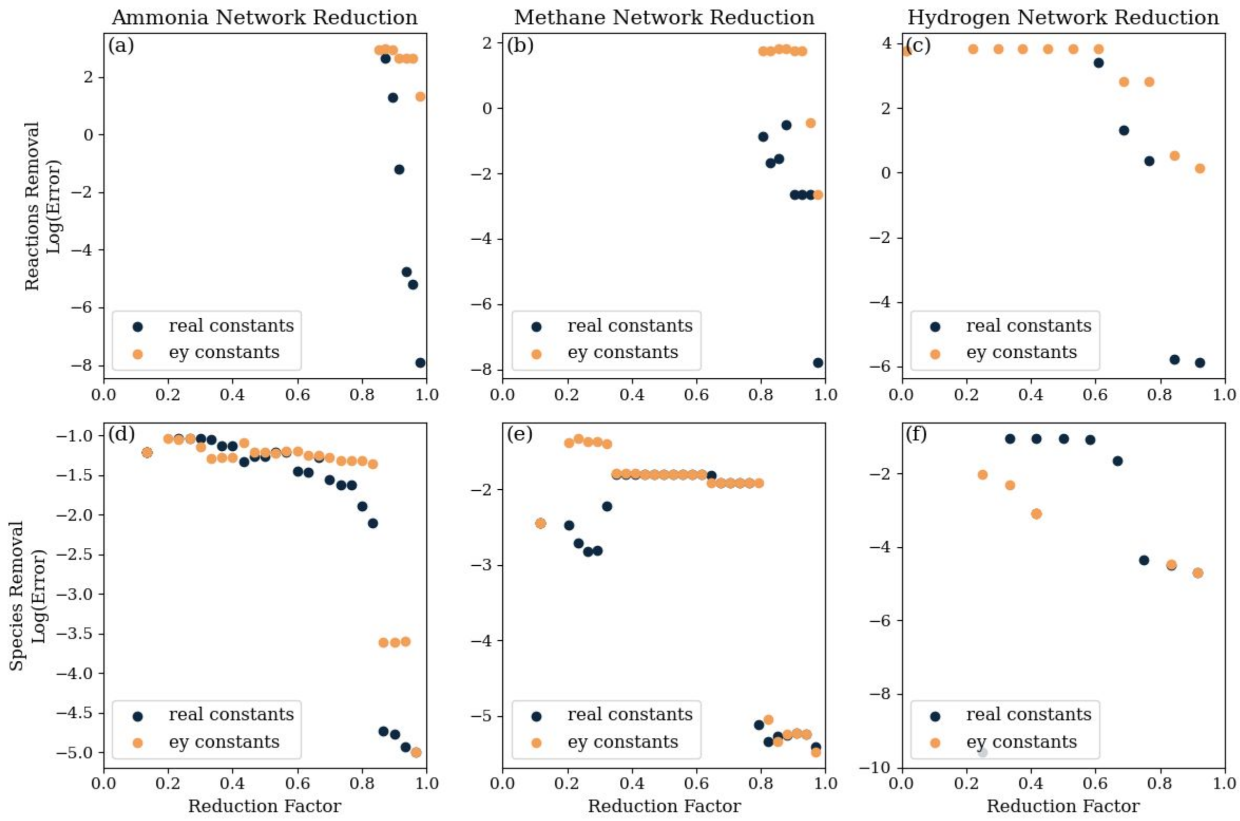

Figure S18: Reduction error as a function of the reduction factor for network reduction based on MolRank scores computed using real and approximated rate constants. Panels (A–C) correspond to reaction removal, where errors are based on reaction rate profiles and the reduction factor is defined as the ratio between the number of reactions in the reduced graph and the initial number. Panels (D–F) correspond to species removal, where errors are based on concentration profiles and the reduction factor is defined analogously.

These results highlight that, while EY-based rate constants can be used within MolRank due to their conservative nature, they do not enhance—and may in fact degrade—the effectiveness of the algorithm as a redundancy metric. In practical terms, this implies that the use of approximated rate constants requires careful calibration of selection thresholds. Specifically, lower MolRank cutoff values must be employed when using EY-based rates in order to achieve reduction quality comparable to that obtained with exact kinetic data. This is further illustrated in Figure S19, where reduction error is shown as a function of the MolRank cutoff: while the overall trends remain similar to those obtained with exact rates, the EY-based reductions consistently exhibit higher errors at the same cutoff values, supporting the need for more conservative threshold selection.

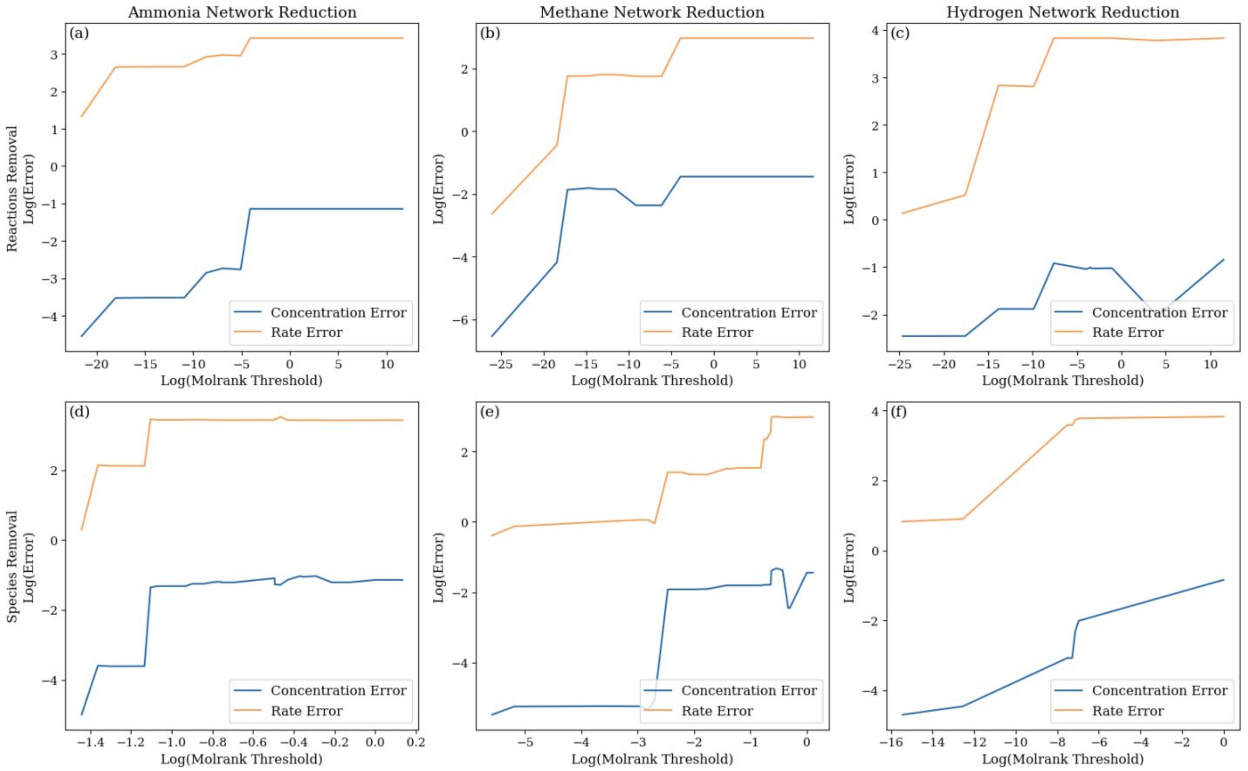

Figure S19: Concentration and rate log-errors vs. MolRank threshold value for removal of (a-c) reactions from ammonia, methane and hydrogen networks, (d-f) species from ammonia, methane and hydrogen networks. With MolRank score based on the Eyring-Polanyi approximated rate constants.

11.2 Comparison to Sensitivity Analysis Metrics

As an additional validation of the MolRank scoring, a complementary sensitivity analysis was performed on the published benchmark mechanisms. Sensitivity analysis is a commonly used tool in reaction network analysis, in which the importance of a reaction is quantified through the response of a selected observable to perturbations in its rate constant. Here, local sensitivities of key intermediate species concentrations with respect to reaction rate constants were evaluated under the same kinetic conditions used throughout this work.

The resulting sensitivity coefficients, for ammonia and hydrogen networks[1], were compared to the corresponding MolRank scores for each reaction. A mild correlation (R^2~0.3) was observed, indicating that both approaches identify overlapping subsets of kinetically influential reactions. It is important to note, however, that the two approaches are not directly equivalent. Sensitivity analysis depends explicitly on the choice of target observables (e.g., specific intermediate species) and reflects local responses under given conditions. In contrast, MolRank provides a global, graph-based ranking that does not require predefined outputs and instead reflects the overall structural and kinetic prominence of reactions within the network. Consequently, no one-to-one correspondence between MolRank scores and sensitivity coefficients is expected.

[1] Methane network was not analyzed due to long computation times

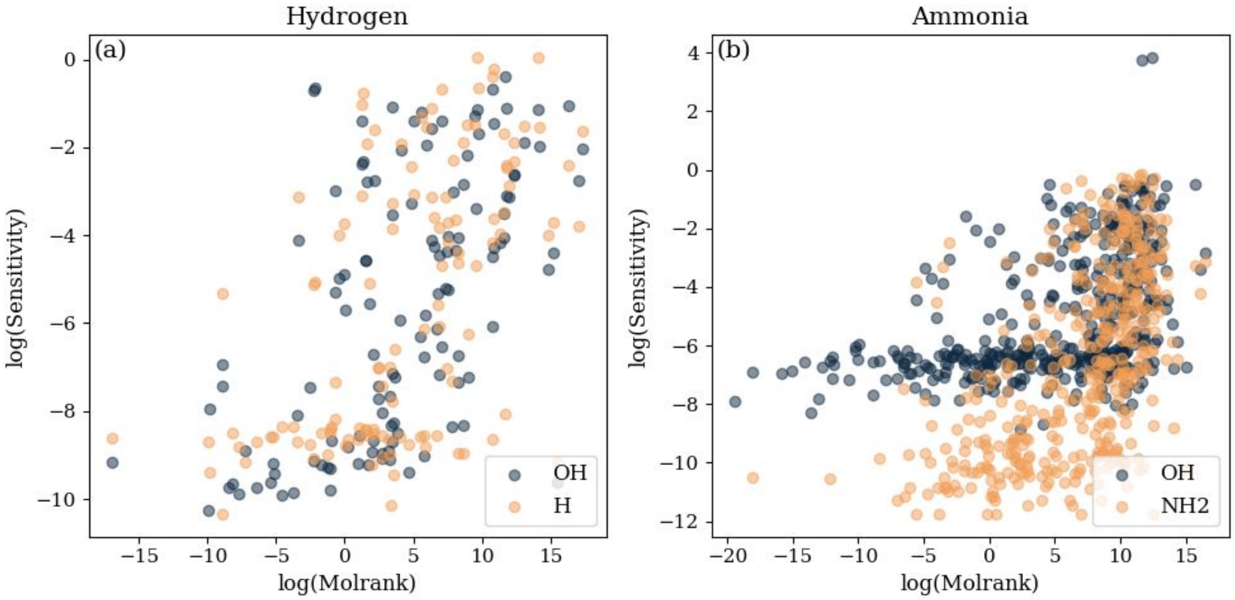

Figure S20: Correlation between Molrank scores of reactions and sensitivity of key intermediates to the reactions' rate constants for (a) hydrogen and (b) ammonia networks.

12 Works Cited

[1] P. M. Zimmerman, "Automated discovery of chemically reasonable elementary reaction steps," *J Comput Chem*, vol. 34, no. 16, pp. 1385–1392, Jun. 2013, doi: 10.1002/jcc.23271.

[2] Y. Kim, J. W. Kim, Z. Kim, and W. Y. Kim, "Efficient prediction of reaction paths through molecular graph and reaction network analysis," *Chem. Sci.*, vol. 9, no. 4, pp. 825–835, 2018, doi: 10.1039/C7SC03628K.

[3] Q. Zhao and B. M. Savoie, "Simultaneously improving reaction coverage and computational cost in automated reaction prediction tasks," *Nat Comput Sci*, vol. 1, no. 7, pp. 479–490, Jul. 2021, doi: 10.1038/s43588-021-00101-3.

[4] C. Robertson, I. Ismail, and S. Habershon, "Traversing Dense Networks of Elementary Chemical Reactions to Predict Minimum-Energy Reaction Mechanisms," *ChemSystemsChem*, vol. 2, no. 4, p. e1900047, Jul. 2020, doi: 10.1002/syst.201900047.

[5] I. Ismail, R. Chantreau Majerus, and S. Habershon, "Graph-Driven Reaction Discovery: Progress, Challenges, and Future Opportunities," *J. Phys. Chem. A*, vol. 126, no. 40, pp. 7051–7069, Oct. 2022, doi: 10.1021/acs.jpca.2c06408.

[6] Greg Landrum *et al.*, *rdkit/rdkit: 2023_09_3 (Q3 2023) Release*. (Dec. 06, 2023). Zenodo. doi: 10.5281/ZENODO.10275225.

[7] G. Goos, *Algorithms ESA 2001: 9th Annual European Symposium á…rhus, Denmark, August 28-31, 2001 Proceedings*, 1st ed. Berlin/Heidelberg: Springer Berlin Heidelberg, 2001.

[8] P. Virtanen *et al.*, "SciPy 1.0: fundamental algorithms for scientific computing in Python," *Nat Methods*, vol. 17, no. 3, pp. 261–272, Mar. 2020, doi: 10.1038/s41592-019-0686-2.

[9] F. Neese, "Software update: The ORCA program system—Version 5.0," *WIREs Comput Mol Sci*, vol. 12, no. 5, p. e1606, Sep. 2022, doi: 10.1002/wcms.1606.

[10] S. Müller, C. Flamm, and P. F. Stadler, "What makes a reaction network 'chemical'?," *J Cheminform*, vol. 14, no. 1, p. 63, Sep. 2022, doi: 10.1186/s13321-022-00621-8.
